# Questions of Mirror Symmetry at the Photoexcited and Ground States of Non-Rigid Luminophores Raised by Circularly Polarized Luminescence and Circular Dichroism Spectroscopy: Part 1. Oligofluorenes, Oligophenylenes, Binaphthyls and Fused Aromatics

**DOI:** 10.3390/molecules23102606

**Published:** 2018-10-11

**Authors:** Michiya Fujiki, Julian R. Koe, Takashi Mori, Yoshihiro Kimura

**Affiliations:** 1 Division of Materials Science, Graduate School of Science and Technology, Nara Institute of Science and Technology (NAIST), 8916-5 Takayama, Ikoma, Nara 630-0036, Japan; mori@tri-osaka.jp (T.M.); yoshi19791024uk@gmail.com (Y.K.); 2 Department of Natural Sciences, International Christian University (ICU), 3-10-2 Mitaka, Tokyo, 181-8585, Japan

**Keywords:** circularly polarized luminescence, circular dichroism, symmetry breaking, parity violation, weak neutral current, tunneling, *Z*^0^ boson, fluorene, camphor, binaphthyl, laser dyes

## Abstract

We report experimental tests of whether non-rigid, *π*-conjugated luminophores in the photoexcited (*S*_1_) and ground (*S*_0_) states dissolved in achiral liquids are mirror symmetrical by means of circularly polarized luminescence (CPL) and circular dichroism (CD) spectroscopy. Herein, we chose ten oligofluorenes, eleven linear/cyclic oligo-*p*-arylenes, three binaphthyls and five fused aromatics, substituted with alkyl, alkoxy, phenyl and phenylethynyl groups and also with no substituents. Without exception, all these non-rigid luminophores showed negative-sign CPL signals in the UV-visible region, suggesting temporal generation of energetically non-equivalent non-mirror image structures as far-from equilibrium open-flow systems at the S_1_ state. For comparison, unsubstituted naphthalene, anthracene, tetracene and pyrene, which are achiral, rigid, planar luminophores, did not obviously show CPL/CD signals. However, camphor, which is a rigid chiral luminophore, showed mirror-image CPL/CD signals. The dissymmetry ratio of CPL (*g*_lum_) for the oligofluorenes increased discontinuously, ranging from ≈ −(0.2 to 2.0) × 10^−3^, when the viscosity of the liquids increased. When the fluorene ring number increased, the *g*_lum_ value extrapolated at [*η*] = 0 reached −0.8 × 10^−3^ at 420 nm, leading to (–)-CPL signals predicted in the vacuum state. Our comprehensive CPL and CD study should provide a possible answer to the molecular parity violation hypothesis arising due to the weak neutral current mediated by the *Z*^0^-boson.

## 1. Introduction

Quantum mechanics (QM) relies on the electromagnetic (*EM*) force mediated by the massless photon (*γ*). QM affords the novel hypothesis that true mirror-image molecules are not observable at stationary states [1,2,3]. In 1927, Hund posed a fundamental question referred to as Hund’s paradox, which concerns the relationship between optical activity and molecular chirality in a symmetrical potential energy double-well (DW). Because of quantum tunneling, mirror-image molecules are described by superposition of the odd- and even-parity eigenstates, |*L*> = 1/√2 (|+> − |−>) and |*R*> = 1/√2 (|+> + |−>) or vice versa [1,2,3]. However, because of the parity-conservation law in energetically equivalent mirror-image molecules, the tunneling time between two local minima separated by a potential barrier (*E*_b_) is inversely proportional to the energy splitting ∆*E*_±_ between the odd- and even-parity eigenstates. When the *E*_b_ value is sufficiently low, the molecules spontaneously oscillate between the |*L*> and |*R*> states, leading to the novel concept of *spatiotemporal chirality*. In tri-substituted phosphines, *P*-R_1_R_2_R_3_, *E*_b_ ≈ 37 kcal mol^−1^ and the tunneling splitting, ∆*E*_±_, is of the order of 10^−17^ eV [2], so the quantum oscillation of optical activity cannot be detected. On the other hand, because the inversion barrier of ammonia due to quantum tunneling is around 5.8 kcal mol^−1^, a ∆*E*_±_ of 0.8 cm^−1^ is observable [4]. Ammonia molecules thus spontaneously oscillate with time, in a temperature-independent flip-flop motion via a tunneling mechanism.

In 1956, Lee and Yang suggested from a theoretical standpoint that the parity conservation law in certain nuclear reactions, due to the weak nuclear force, was not yet proved [4]. Wu et al. and other workers verified the parity violation (*PV)* hypothesis at the sub-atomic level in a series of *β*^−^- and *β*^+^-decay experiments of several radioactive sources [5,6,7,8,9,10]. These results stimulated other work, resulting in what is known as the electroweak (*EW*) theory, to unify the *PV* weak and parity-conserving *EM* forces in the 1960s [11]. In 1983, massive charged *W*^±^ (~80 GeV) bosons and the neutral *Z*^0^ boson (~91 GeV) were indeed detected at CERN [12,13,14,15].

*PV* effects at sub-atomic level were successfully detected during spontaneous nuclear emission processes. The *β*^−^*-*decay of ^60^Co→^60^Ni mediated by the *W*^−^ boson radiates a left-handed electron (0.317 MeV), left-handed circularly polarized *γ*-rays (1.173 GeV and 1.332 GeV) and a right-handed anti-neutrino (<2 eV) [16]; conversely, the *β*^+^-decay in ^58^Co→^58^Fe by the *W*^+^ boson releases a right-handed positron (0.475 MeV), a right-handed circularly polarized *γ*-ray (0.811 GeV) and a left-handed neutrino [12]. 

The *EW* force mediated by the *Z*^0^ boson further predicated the induction of hidden optical activity in all atoms because of left-handed *Z*^0^ [17,18]. Actually, optical activity of atomic vapors (Pb, Tl, Bi, Cs, Yb and others) [19,20,21,22,23,24,25,26] and solid metal (^117^Sn) [27] has been detected as an atomic parity violation (*APV*) [19,20,21,22,23,24,25,26,27]. The optical activity of the atomic vapors has a dissymmetry ratio (*g*) ≈ −10^−7^ associated with an anapole moment in the visible and near-IR regions characterized by optical rotation dispersion (ORD) and electric field-induced absorption and polarized luminescence (CPL) spectra [18,19,20,21,22,23,24,25,26]. Compared to electric field-induced absorption experiments, the profound *APV* effects of Cs and Tl vapors in the photoexcited state at the forbidden transitions are more sensitively detectable as CPL and PL signals [20,22,23] and also that of Yb vapors was boosted by two orders of magnitude using AC-Stark induced second harmonic transitions under a static magnetic field [26].

Additionally, since the early 1970s and 1980s, several theoreticians have invoked the molecular parity violation (*MPV*) hypothesis from the viewpoint of hierarchical matter world in our Universe: the parity-violating weak neutral current (*PV-WNC*) in the electron–nuclei interactions mediated by the *Z*^0^-boson stabilizes one of the mirror-image molecules and conversely destabilizes the other [1,2,28,29,30,31,32,33,34,35,36,37,38,39]. The *MPV* hypothesis predicts that conventional mirror-image molecules are thus no longer enantiomers and should behave as diastereomers due to parity violating energy shifts (*PVES*), in which +*E*_PV_ for one and −*E*_PV_ for another, now popularly called parity violating energy difference (*PVED*), ∆*E*_PV_ or simply *E*_PV_ are evident. The hypothesis definitively contradicts modern stereochemistry as described in textbooks and lecture notes. However, most *MPV* theories [1,2,28,29,30,31,32,33,34,35,36,37,38,39,40,41,42,43,44,45,46,47,48,49,50] have estimated that the value of *E*_PV_ between mirror-image molecules are vanishingly small: of the order of 10^−8^–10^−14^ kcal mol^−1^. These *E*_PV_ values are equivalent to an excess of one *l*- (or *R-*) molecule in a racemic mixture of 10^11^–10^17^ molecules (10^−9^–10^−15^% *ee*), therefore, beyond the detection capabilities of ordinary UV-visible, IR and NMR spectrometers and chromatographic instruments available in typical laboratories. Even if the *MPV* hypothesis is valid, one cannot at all recognize the difference between enantiomers and agrees that the enantiomers obey the stereochemistry descriptions in the textbooks. However, several amplification scenarios of *PVED* have been postulated, leading to spectroscopically detectable levels.

For example, Yamagata first proposed an accumulation model in which the faint *E*_PV_ is linearly amplified by the association of molecules into chain-like polymers and/or solid crystals [28]. Harris and Stodolsky assumed that the *PC*-*EM*-originating quantum oscillation of hypothetical chiral molecules in a DW is slightly influenced by *PV-*originating oscillation [34]. Other theoretical models of realistic molecules were discussed by Hegstrom et al. (twisted ethylene) [35], Macdermott and Hegstrom (ammonia-like molecules) [47], Mason and Tranter (glycine and polyglycine) [36] and Kikuchi et al. (*n*-alkanes) [43]. Salam predicted that the second-order phase-transition of a molecular system, as exemplified by amino acids, spontaneously shows a handed mirror symmetry breaking (MSB) at *T*_c_ ~250 K [41]. Quack showed that high-resolution ro-vibrational spectroscopy is useful to detect the *PVED* of around 40 realistic and hypothetical molecules [2,48,49]. Schwerdtfeger et al. showed that subtle *E*_PV_ effects are enhanceable with the help of relativistic effects in heavier atoms [47,50]. P. Bargueño et al. simulated decoherence behaviors of dissipative chiral molecules in two-level systems, when tunneling and parity violation effects are opposed [51].

Quack et al. [46,49] and Macdermott and Hegstrom [48] discussed three representative cases of (i) *E*_PV_ >> *E*_±_, (ii) *E*_PV_ << *E*_±_ and (iii) *E*_PV_ ~ *E*_±_ in several molecules. The ultra-small, handed-signed *E*_PV_ of rigid enantiomers cannot interfere with *E*_±_ value. Quack tabulated *E*_PV_ and *E*_±_ values of twenty non-rigid rotamers and showed that the sign of *E*_PV_ in non-rigid XZ–ZX type rotamers (X = H, D, T, Cl and Z = O, S, Se, Te) alters, depending on the rotatable angles and also that in XO–OX molecules (X = H, D, T, Cl), *E*_PV_ << *E*_±_. [49] Although the *E*_PV_ and *E*_±_ values are susceptible to the nature of the rotamers, the former changes by a factor of 10^5^ while the latter changes by a factor of 10^25^. This estimation implies an increase in rotational barrier height of XZ–ZX rotamers. Actually, among the XZ–ZX rotamers, only T_2_S_2_ satisfies the *E*_PV_ ~ *E*_±_ condition [46]. The rotational barrier of HO–OH is rather high: around 7.5 kcal mol^−1^. To interfere efficiently between *E*_PV_ and *E*_±_, the *E*_PV_~*E*_±_ criterion using non-radioactive substances should be designed experimentally because radioactive T is not available.

So far, several experimental groups reported the validity of the MSB hypothesis by seeking subtle differences in physical and optical properties between enantiomeric species; for example, high-resolution vibrational and ro-vibrational spectroscopy of rigid chiral organic and inorganic molecules in a vacuum [52,53], spontaneous crystallization of coordination compounds including heavy metal ions [54], single crystals of *d*-/*l*-amino acids [55], helix-coil transition characteristics of oligo-*d*-/*l*-amino acids [56], α-helix→3_10_-helix transition characteristics of poly-(*d*-/*l*-)-glutamic acids [57], 7_3_-helix→7_3_-helix transitions characteristics of Si–Si bond polymers [58,59,60] and more recently, anomalies in thermal and NMR relaxation properties of metal-organic frameworks [61,62]. The possibility of detecting a *D-L* preference between enantiomers under thermal equilibrium conditions is beyond doubt, yet definitive experimental answers have remained elusive because of the stringent requirements for very specific mirror-image molecules satisfying 100% *ee* purity in the ground (*S*_0_) state.

By learning from successful *β*^±^-decay and *APV* radiation experiments [6,7,8,20,22], we postulated that non-rigid *π*-conjugated luminophores in symmetrical DW/multiple-wells (MW) with lower *E*_b_ values in the lowest photoexcited (*S*_1_) state at the allowed transitions allow for possible detection the *MPV* effect. Aiming at verifying our idea, we tested several non-rigid luminophores including oligofluorenes (up to seven repeating units), linear and cyclic oligo-*p*-arylenes (up to twelve repeating units), C_2_-symmetrical binaphthyls and fused aromatics (three acenes and pyrenes) carrying rotatable side groups. To control experimentally the tunneling barrier *E*_b_ in these luminophoric systems at the *S*_1_/*S*_0_ states, we chose various achiral liquids, including linear-/branched alkanes, linear-/branched alkanols, haloalkanes, linear-/cyclic-ethers and others. The viscosity [*η* in *cP*] is broadly tunable, ranging from 0.21 to 71.0 at 20–25 °C [63,64,65]. The solvent viscosity plays a role in the *continuous* control of the *E*_b_ value of the tunneling potential surface of non-rigid molecules [48,49,51] at the *S*_1_/*S*_0_ states, enabling fulfillment the *E*_±_ ~ *E*_pv_ criteria.

Without exception, all the non-rigid fluorophores showed negative-sign CPL signals in the UV-visible region, suggesting that energetically inequivalent, non-mirror image structures at the *S*_1_ state are temporarily generated. For comparison, four fused aromatic fluorophores did not show clear CPL signals. The non-rigid fluorophores boosted progressively but *discontinuously* in a quantized behavior while keeping negative-sign CPL spectra in the range of *g*_lum_ = −0.2 × 10^−3^ to −2.0 × 10^−3^ in response to the viscosity of the liquids. In a series of oligofluorenes, the *g*_lum_ value at zero viscosity was extrapolated to −0.8 × 10^−3^ at 420 nm when more than 7 fluorene rings are fused. This non-zero *g*_lum_ value suggested that the photoexcited oligofluorenes emit (–)-sign CPL signals in a vacuum, originating from the hypothesized *PV*-*WNC* coupled with the ring-current of the aromatics under conditions of zero magnetic field.

## 2. Results

The handed electron–nuclei interactions force enables CPL-silent/CD-silent molecules into CPL-active/CD-active ones at the *S*_1_/*S*_0_ states. Although heavier atoms, like Si, Ge, Sn, Pb, Se, Te, Fe, Ru, Re, W, Os and U, are involved in the luminophores, *E*_PV_ may be boosted by the *V*_SO_ (∝ *Z*^2^) law. However, luminescent species might be phosphorescent with an inefficient quantum yield (PLQY, typically, <0.01) and is difficult to detect as CPL/PL signals in an excellent S/N ratio. Therefore, we focused on fluorophores comprising lighter atoms because of an efficient PLQY, typically, 0.1–0.9. The values of the spin-orbit interaction (*ζ*) for lighter atoms (C, N, O, F, S) are considerably large *ζ* values such that 0.1, 0.2, 0.4, 0.7, 1.0 kcal mol^−1^, respectively [66]. These fluorophores are utilized as laser dyes, highly emissive dyes, biomarkers and scintillators. If huge numbers (>10^10^–10^16^) of the non-rigid enantiomeric fluorophores in a cuvette are photoexcited macroscopically and simultaneously, the ultra-tiny *ee* or faint *E*_PV_ of an isolated molecule may be greatly boosted to the detectable level, according to the linear amplification model [28] and/or the highly cooperative phase transition models at the far-from, non-equilibrium, open-flow *S*_1_ state [41].

Alternatively, when Kasha’s rule in Jablonski diagram is considered [66,67], fluorescence should occur from only ‘the lowest vibrionic *S*_1_ state’ during the courses of non-radiative, ro-vibrational/translational processes even if the luminophores are photoexcited at *S*_2_ and higher *S*_n_ states. Possible radiation scenarios are classified to four cases.

The inherently rigid achiral luminophores do not emit CPL signals and do not reveal CD signals.

The rigid chiral luminophores involve point chirality and/or axial chirality, that act as parity-conserving strong chiral electromagnetic (*EM*) force at the *S*_1_/*S*_0_ states. The rigid chiral luminophores at the *S*_1_ state adopt the similar geometry of the corresponding *S*_0_ state due to minimal reorganization processes in a highly symmetrical DW, sign and magnitude of CPL between enantiomers should be mirror-image.

When non-rigid CD-silent enantiomers in a symmetrical DW at the *S*_1_ state coexist equally, the enantiomers may rapidly oscillate between *L*- and *R*-states with time at the *S*_1_ state, resulting in time-averaged null-CPL signals.

If the non-rigid enantiomers turn into a dissymmetrical DW at the *S*_1_ state by quitting the oscillating fashion, only (+)- or (–)-sign CPL signals are detectable *via* noticeable handed rotational and/or flip-flop motions.

### 2.1. Rigid Achiral Planar π-Conjugated Luminophores

**Chart 1:**
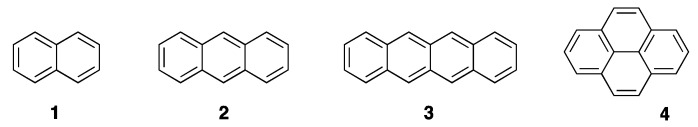
Chemical structures of *D*_2h_-symmetrical fused aromatics: naphthalene (**1**), anthracene (**2**), tetracene (**3**) and pyrene (**4**).

**Figure 1:**
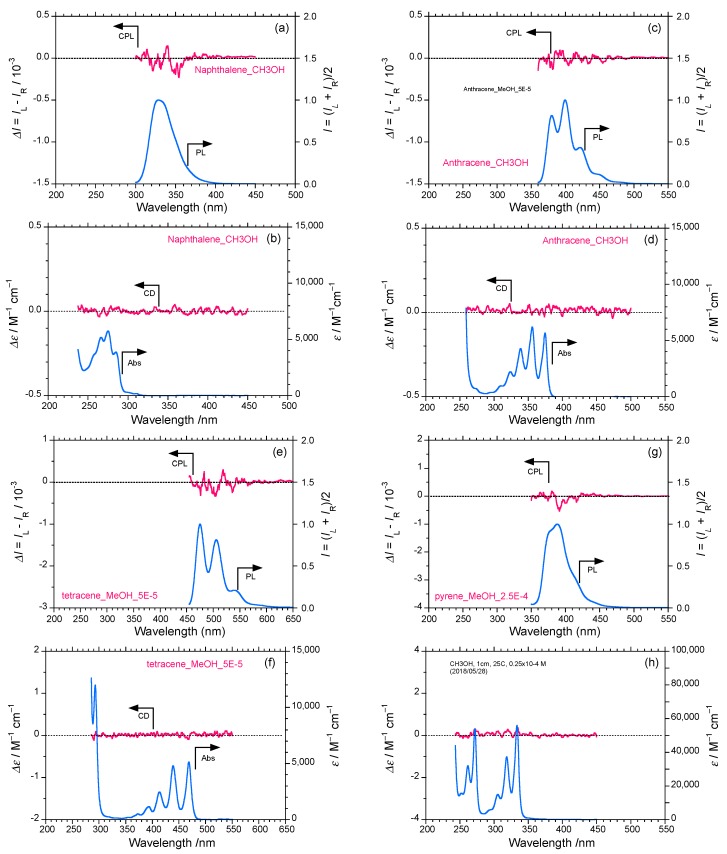
CPL/PL and CD/UV-vis spectra of *D*_2h_-symmetrical fused aromatics in methanol at room temperature. (**a**) CPL/PL spectra excited at 270 nm and (**b**) CD/UV-vis spectra of **1**. (**c**) CPL/PL spectra excited at 310 nm and (**d**) CD/UV-vis spectra of **2**. (**e**) CPL/PL spectra excited at 420 nm and (**f**) CD/UV-vis spectra of **3**. (**g**) CPL/PL spectra excited at 320 nm and (**h**) CD/UV-vis spectra of **4**. Measurement conditions: path length: 10 mm, cylindrical cuvette, conc.: (2.5–5.0) × 10^−5^ M.

Firstly, to ensure neutrality in CPL- and CD-signals of achiral *D*_2h_ symmetrical *π*-conjugated aromatic compounds that involve naphthalene (**1**), anthracene (**2**), tetracene (**3**) and pyrene (**4**) (Chart 1) without substituents in the *S*_1_ and *S*_0_ states, we measured the CPL- and CD-spectra in a low viscosity methanol ([*η*] = 0.55 *cP*) and several solvents. Although artifact-free precision measurements are serious concerns of single 50-KHz PEM CPL and CD spectrometers [68,69,70], no detectable CPL- and CD-signals at the corresponding PL and UV-visible spectra were confirmed for **1** (Figure 1a,b), **2** (Figure 1c,d), **3** (Figure 1e,f) and **4** (Figure 1g,h). Our CPL (JASCO CPL-200) and CD (JASCO J-820) spectrometers guaranteed the null CD and CPL signals of **1**–**4**.

### 2.2. Rigid Planar π-Conjugated Luminophores Bearing Phenyl and Phenylethynyl Rotors

**Chart 2:**
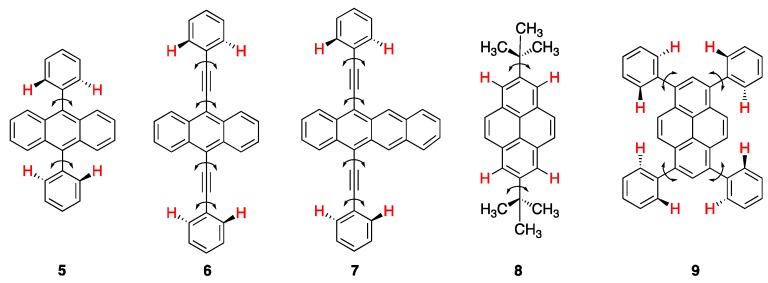
Chemical structures of fused aromatics bearing rotatable substituents: 9,10-diphenylanthracene (**5**), 9,10-bis(phenylethynyl)anthracene (**6**), 5,12-bis(phenylethynyl)- tetracene (**7**), 2,7-di-*tert*-butylpyrene (**8**) and 1,3,6,8-tetraphenylpyrene (**9**).

**Figure 2:**
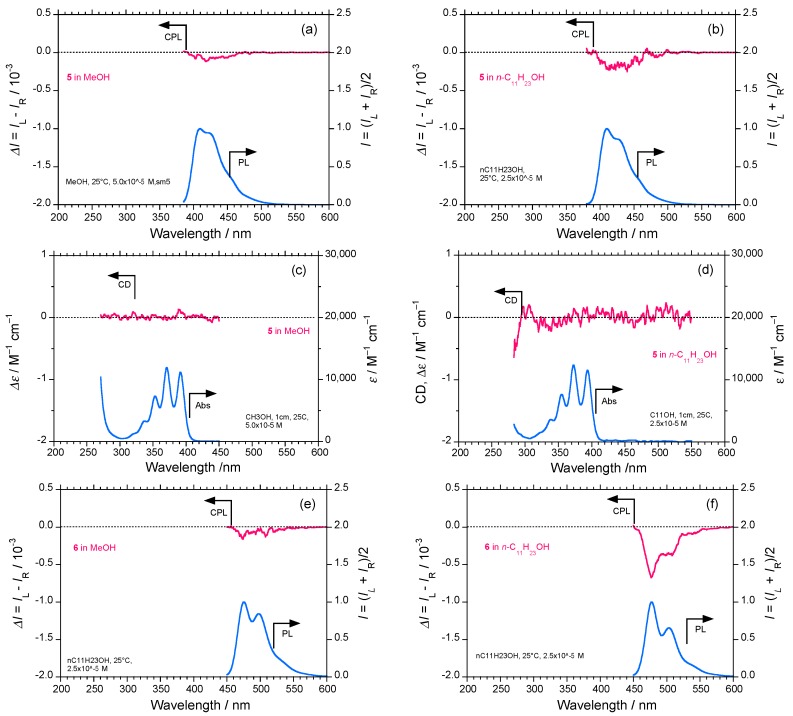

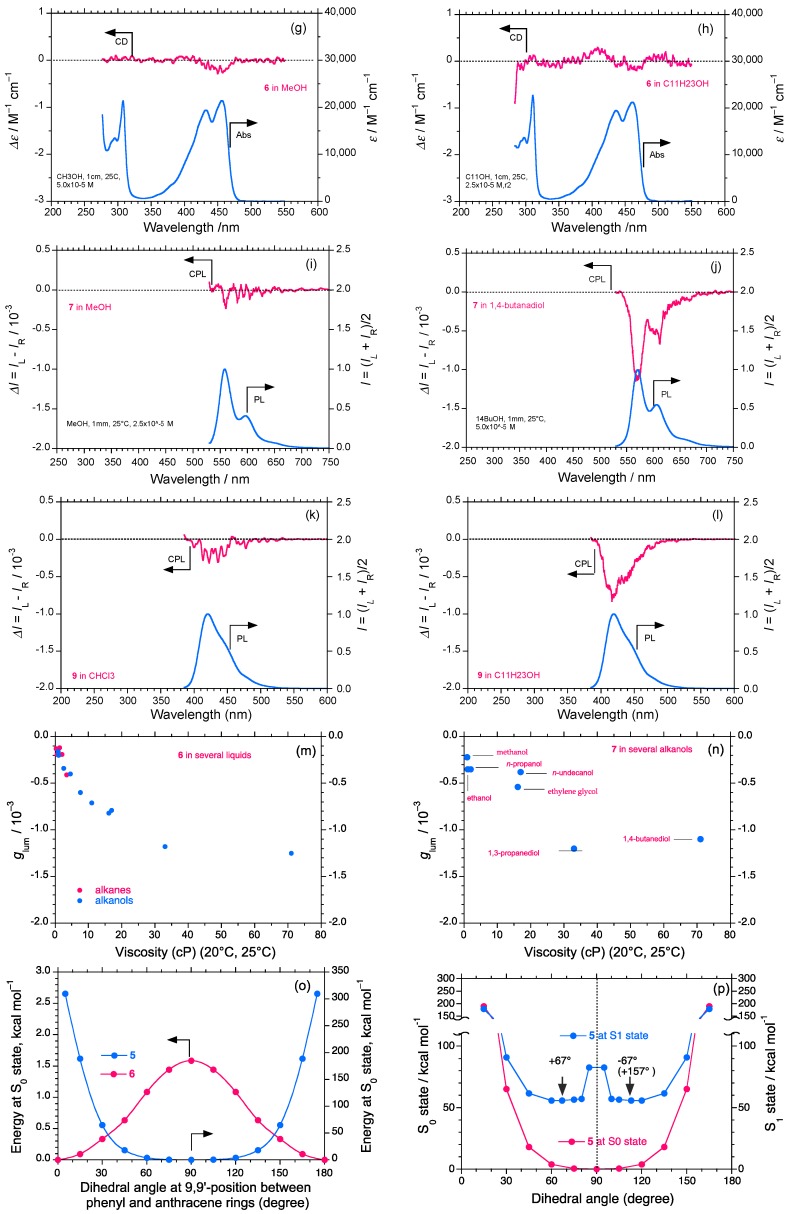
CPL/PL and CD/UV-vis spectral characteristics of pseudo-*D*_2h_-symmetrical fused aromatics carrying multiple rotatable substituents in various liquids at room temperature. (**a**) CPL/PL spectra of **5** excited at 355 nm and (**c**) CD/UV-vis spectra in methanol. (**b**) CPL/PL spectra of **5** excited at 355 nm and (**d**) CD/UV-vis spectra in *n*-undecanol. (**e**) CPL/PL spectra of **6** excited at 420 nm and (**g**) CD/UV-vis spectra in methanol. (**f**) CPL/PL spectra of **6** excited at 420 nm and (**h**) CD/UV-vis spectra *n*-undecanol. CPL/PL spectra of **7** excited at 500 nm in (**i**) methanol and (**j**) 1,4-butanediol. CPL/PL spectra of **9** excited at 355 nm in (**k**) chloroform and (**l**) *n*-undecanol. (**m**) The *g*_lum_ value (in 10^−3^) at 475 nm of **6** as a function of viscosity of achiral liquids consisting of alkanes and alkanols; *n*-pentane, −0.13, *n*-octane −0.19, *n*-decane −0.17, *n*-dodecane −0.12, *n*-tetradecane −0.19, *n*-hexadecane −0.41, methanol −0.16, ethanol −0.20, *n*-butanol −0.34, *n*-hexanol −0.40, *n*-octanol −0.60, *n*-decanol −0.71, *n*-undecanol −0.79, ethylene glycol −0.82, 1,3-propanediol −1.18, 1,4-butanediol-1.25. (**n**) The *g*_lum_ value (in 10^−3^) of **7** as a function of viscosity of achiral liquids. Measurement conditions: path length: 10 mm, cylindrical cuvette, conc.: (2.5–5.0) × 10^−5^ M at room temperature. (**o**) Energy of **5** and **6** optimized at *S*_0_ state as a function of dihedral angle of two phenyl moieties at 9,10-position of anthracene. (**p**) Energy of **6** optimized at *S*_0_ and *S*_1_ states as a function of dihedral angle of two phenyl moieties at 9,10-positions of anthracene. These calculations were performed by DFT method (B3LYP functional with 6-31Gd basis set).

On the other hand, pseudo-*D*_2h_ symmetrical frameworks bearing rotatable phenyl groups and phenylethynyl groups (**5**–**9**, Chart 2) showed (–)-sign CPL signals at the corresponding PL bands in methanol and other solvents (Figure 2 and Figure S1 Supplementary Materials (SM)). Molecules **5** and **6**, corresponding to **1** bearing two phenyl groups and two phenylethynyl groups in the 9,10-positions, respectively, showed weak vibronic CPL bands in methanol (Figure 2a,e). Evidently, the CPL signals of **5** and **6** are significantly boosted in the more viscous solvents *n*-undecanol (16.95 *cP*) and 1,4-butanediol (71.0 *cP*) (Figure 2b,f and Figure S1a in SM)).

It is notable that the CPL signals of **6** in *n*-undecanol are more obvious than those of **5** in *n*-undecanol, though the rotational barrier of the phenylethynyl group should be smaller than that of phenyl group. We observed a very weakly (–)-sign CD signal for **6** in methanol on the order of *g*_abs_ ~ −1 × 10^−5^ at 456 nm (Figure 2g), while **6** in *n*-undecanol a clear CD signal is not obvious (Figure 2h). Subtle alteration of the phenyl and phenylethynyl groups in the peripheral 9,10-positions of **2** may affect these CPL and/or CD characteristics. Similarly, molecule **7**, which is a derivative of **3** carrying phenylethynyl groups in 5,12-positions, showed clearly vibronic CPL signals at the corresponding PL bands in 1,4-butanediol (Figure 2j), although the signals in methanol were very weak (Figure 2i). The CD signal of **7** in methanol is not obvious (Figure S2b in SM).

These noticeable solvent dependent CPL characteristics led us to plot the *g*_lum_ values of **6** and **7** as a function of the viscosity of *n*-alkanes and alkanols (Figure 2m,n). When [*η*] > 30 *cP*, the *g*_lum_ values of **6** and **7** progressively approach to −1.2 × 10^−3^. These *g*_lum_–viscosity relationships might be the key in order to discuss the mirror symmetry breaking of semi-rigid and non-rigid luminophors at the *S*_1_ state.

To further verify the substituent effects, we tested the CPL/PL spectral characteristics of two pyrene derivatives (**9**) carrying four phenyl groups in the 1,3,6,8-positions of **4** and for comparison, **8** carrying two *tert*-butyl groups in the 2,7-positions of **4**. Similar to the case of **5**, molecule **9** in *n*-undecanol clearly revealed (–)-sign CPL signals, which were only weakly evident in chloroform (Figure 2k,l). Molecule **8** in *n*-C_6_H_14_ ([*η*] = 0.30 *cP*) revealed derivative-like vibronic CPL signals (Figure S1c in SM). The steric repulsion between multiple protons on the phenyl rings in **5** and **9** (marked in red, Chart 2) and multiple protons in their fused aromatic frameworks may efficiently induce certain handed twisted conformations at the *S*_1_ states. CD signals of **8** in methanol and **9** in methanol and *n*-undecanol are not obvious (Figure S1d–f) in SM). Further CD measurements confirmed no detectable signals for **8** or **9** in the range 400–500 nm in ethanol, *n*-propanol, 1,3-propanediol or 1,4-butanediol (Figure S1f in SM).

To test the degree of steric repulsion, Gaussian09 calculations (DFT, B3LYP functional at 6-31G (d, *p*) level) of **5** and **6** at the *S*_0_ and *S*_1_ states were employed as a function of the dihedral angle (*θ*) between the anthracene and phenyl rings. Molecule **5** prefers a perpendicular geometry at *θ* = 90°, while, **6**, conversely, prefers a coplanar geometry with *θ* = 0°/180° (Figure 2o). However, **5** at the *S*_1_ state has two global minima, at +67° and +157° (–67°), indicating that the anthracene and phenyl rings prefer a tilted aspect with left- and right-twisted geometries (Figure 2p). However, **6** at the *S*_1_ state, prefers a coplanar geometry with *θ* = 0°/180° (not shown, similar to Figure 2o). If the two twists exist equally at the *S*_1_ state, CPL signals are not detectable. If the two twists exist due to energetical inequality at the *S*_1_ state, CPL signals are detectable.

### 2.3. Rigid and Non-Rigid Chiral Luminophores

**Chart 3:**
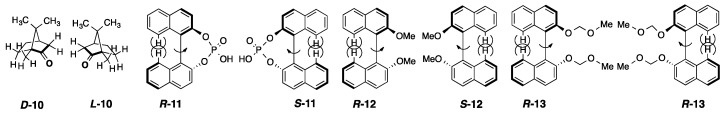
Chemical structures of rigid C_1_-symmtric aliphatic ketone and semi-rigid pseudo-C_2_-symmtric binaphthyl derivatives: *D*- and *L*-camphor (**10**), (*S*)- and (*R*)-1,1’-binaphthyl-2,2’-diyl hydrogen phosphate (**11**), (*S*)- and (*R*)-2,2’-dimethoxy-1,1’-binaphthyl (**12**) and (*S*)- and (*R*)-2,2’-bis(methoxymethoxy)-1,1’-binaphthyl (**13**).

To confirm whether rigid chiral luminophores at the *S*_0_ and *S*_1_ states reveal mirror image CD and CPL spectra in UV-visible regions, we tested enantiomers of *C*_1_-symmetrical camphor (*D*-10 and *L*-10) and three kinds of pseudo-*C*_2_-symmetrical BINOL derivatives with axial chirality (*R*-11 and *S*-11, *R*-12 and *S*-12 and *R*-13 and *S*-13) (Chart 3). Camphor is a representative chiral bicyclic ketone and often used as a chiroptical standard for CPL, CD and VCD spectrometers as it is a naturally occurring chiral substance with a high enantiopurity [70,71]. Also, *R*-11 and *S*-11 are known to emit CPL signals with equal magnitude and the opposite sign as a result of the lack of rotational freedom between the two naphthyl rings restricted by hydrogen phosphophate linkage [72,73,74,75]. However, it is noting that *R*-12/*S*-12 and *R*-13/*S*-13 retain rotational freedom at the 1,1’-axial positions between the two naphthyl rings [72,74].

**Figure 3:**
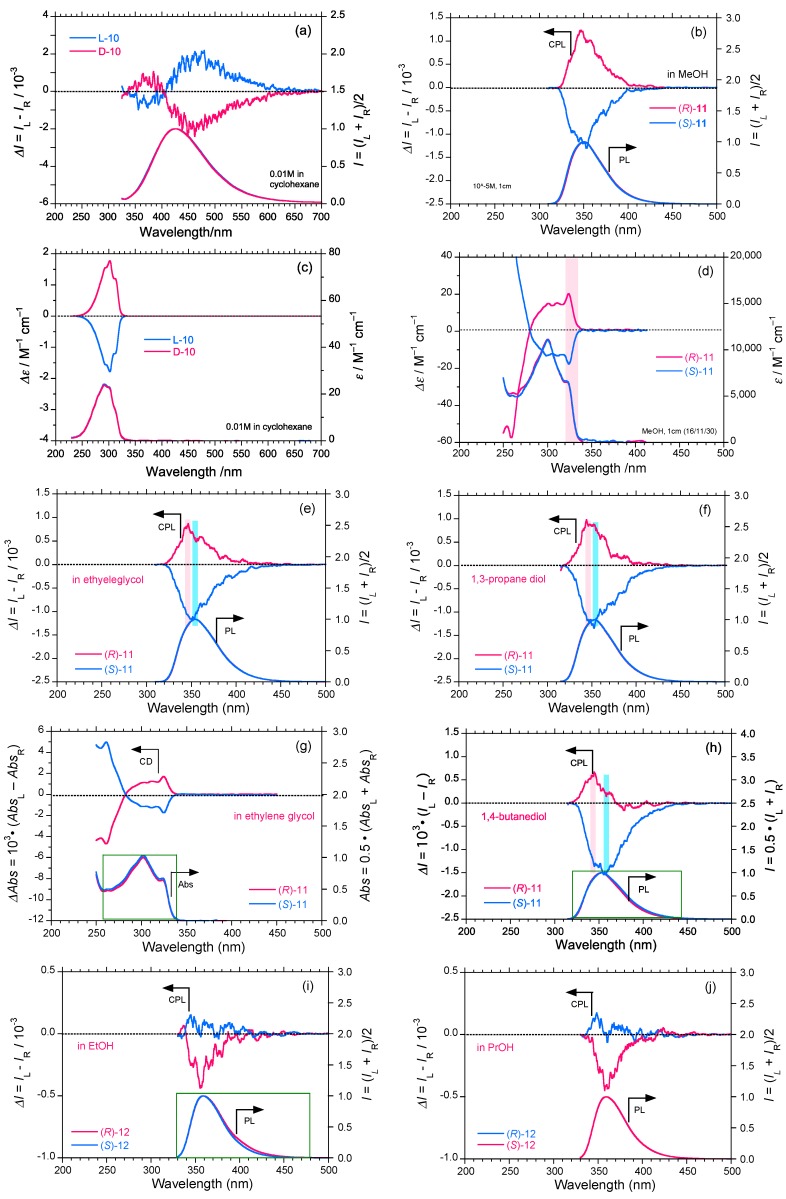

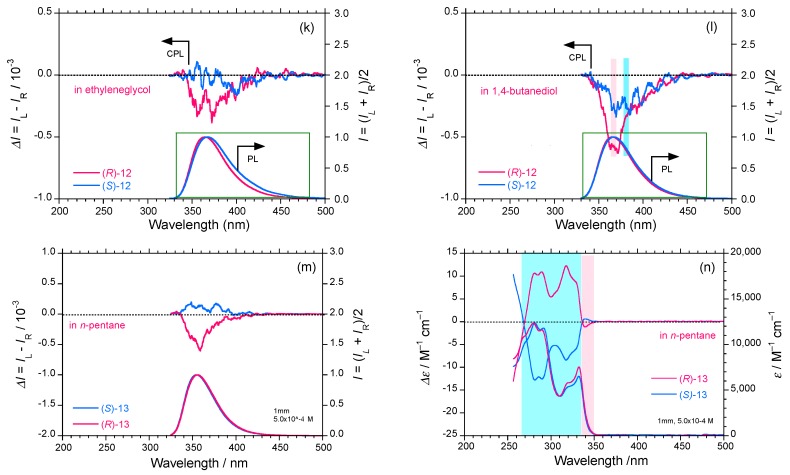
CD/UV-vis and CPL/PL spectra of rigid bicyclic camphor, rigid binaphthol derivatives and semirigid binaphthol derivatives allowing pivotal motion in various solvents at room temperature (path length: 10 mm, cylindrical cuvette, [conc]_0_ = (2–5) × 10^−4^ M: (**a**) CPL/PL spectra of *D*-10 and *L*-10 excited at 295 nm and (**c**) CD/UV-vis spectra in cyclohexane; (**b**) CPL/PL spectra of *R*-11 and *S*-11 excited at 290 nm and (**d**) CD/UV-vis spectra in methanol; CPL/PL spectra of *R*-11 and *S*-11 excited at 290 nm (**e**) in ethylene glycol, (**f**) in 1,3-propanediol and (**h**) in 1,4-butanediol; (**g**) CD/UV-vis spectra of *R*-11 and *S*-11 in ethylene glycol; CPL/PL spectra of *R*-12 and *S*-12 excited at 295 nm (**i**) in ethanol, (**j**) in *n*-propanol, (**k**) in ethylene glycol and (**l**) in 1,4-butanediol; (**m**) CPL/PL spectra *R*-13 and *S*-13 excited at 295 nm and (**n**) CD/UV-vis spectra in *n*-pentane.

The rigid luminophores, *D*-10/*L*-10, in cyclohexane revealed ideal mirror image profiles of vibronic CD and non-vibronic bisignate CPL spectral line shapes with opposite sign and equal magnitude (Figure 3a,c) [70,71]. Similarly, *R*-11/*S*-11 in lower viscosity solvents (*n*-pentane (0.21 *cP*), methanol (0.55 *cP*) and chloroform (0.55 *cP*)) revealed mirror image vibronic CD and vibronic CPL spectral profiles with opposite sign and equal magnitude (Figure 3b,d and Figure S2a,b in SM). Thus, the CPL and CD spectrometers afford reliable evidence for the measurement of ideal mirror-image CD and CPL spectral sets between enantiomers of rigid chiral emitters in lower viscosity solvents at the *S*_1_ and *S*_0_ states. However, *R*-11/*S*-11 in higher viscosity solvents (ethylene glycol (16.1 *cP*), 1,3-propanediol (33.0 *cP*) and 1,4-butanediol (71.0 *cP*)) [64] revealed non-mirror image CPL spectra (Figure 3e,f,h). As the viscosity increased from 16 to 71 *cP*, the absolute magnitude of the CPL of (*R*)-11 at ~350 nm weakens. Moreover, CPL *λ*_ext_ wavelengths (pink and sky-blue bars) between (*R*)- and (*S*)-11 progressively tended to differ from each other. Noticeably, the corresponding CPL spectra between (*R*)- and (*S*)-11 in 1,4-butanediol differ significantly from each other, indicating the absence of enantiomers in the *S*_1_ state (green box in Figure 3h). Also, the corresponding UV-visible spectra of (*R*)- and (*S*)-11 in ethylene glycol differ from each other, indicating the absence of enantiomers in the *S*_0_ state (green box in Figure 3g).

Similarly, although *R*-12/*S*-12 in lower viscosity solvents such as *p*-dioxane (1.10 *cP*), chloroform, *n*-pentane and methanol showed CPL spectra with near mirror image profiles but with subtle differences associated with vibronic bands (Figure S2c–f in SM), *R*-12/*S*-12 in ethanol (1.09 *cP*), *n*-propanol (1.96 *cP*) and ethylene glycol reveals the absence of mirror image CPL spectra (Figure 3i–k) and in 1,4-butanediol (71 *cP*) showed typically (–)-sign CPL spectra associated with different wavelengths (Figure 3l), whilst the CPL *λ*_ext_ wavelengths (pink and sky blue bars) between (*R*)-11 and (*S*)-11 differ from each other (Figure 3l). These non-mirror image CPL spectral characteristics can be recognized from the inconsistent PL spectral profiles between *R*-12 and *S*-12 in ethanol, ethylene glycol, 1,3-propanediol and 1,4-butanediol (green boxes in Figure 3i,k,l and Figure S2g in SM.

On the other hand, *R*-12 and *S*-12 at the *S*_0_ state reveal ideal mirror image CD spectra in these solvents, indicating adoption of mirror-image geometries regardless of the solvents (Figure S3h–j) in SM). It is noteworthy that the longest wavelength CD Cotton effects of *R*-11 and *S*-11 are the opposite of the corresponding *R*-12 and *S*-12 (Figure 3d vs. Figure S3h–j in SM). This apparent inversion phenomenon is likely to arise from alteration in dihedral angle between the two naphthyl rings at the S_1_ state [72,73,74,75]. Actually, *R*-12 showed a (+)-sign CPL spectrum, while *R*-11 showed a (–)-sign CPL spectrum. *S*-12 in 1,4-butanediol favored a (–)-sign CPL spectrum. Similarly, although *R*-13 and *S*-13 at the *S*_0_ state had nearly ideal mirror image CD spectra (Figure 3n), even in *n*-pentane, they no longer had mirror image CPL spectra (Figure 3m).

Thus, semirigid chiral BINOL derivatives, *R*-11/*S*-11 and non-rigid chiral BINOL derivatives, *R*-12/*S*-12 and *R*-13/*S*-13 at the *S*_1_ states showed no mirror image relationship between their CPL spectra, even though mirror image CD spectra were observed at the *S*_0_ states. This anomaly should arise from rotational freedom at the *S*_1_ state between the two naphthyl rings regardless of solvent viscosity, similar to the cases of 5, 6, 7 and 9.

### 2.4. Linear and Cyclic Oligo-p-Phenyl Derivatives without and with Rotatable Alkyl Substituents

**Chart 4:**
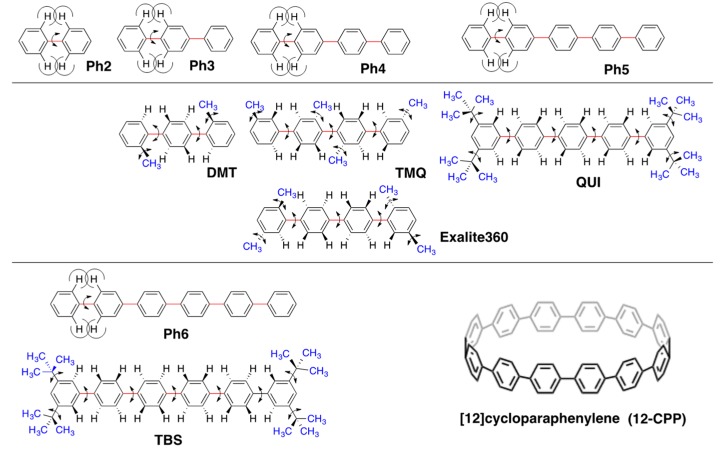
Chemical structures of non-rigid linear and cyclic oligo-*p*-phenyls: biphenyl (Ph2), *p*-terphenyl (Ph3), *p*-quaterphenyl (Ph4), *p*-quinquephenyl (Ph5), *p*-sexiphenyl (Ph6), 2,2″-dimethyl-1,1′:4′1″-terphenyl (DMT), 2″,3,3′,3‴-tetramethyl-1,1′:4′1″:4″,1‴-quaterphenyl (TMQ), 2,5,2‴,5‴-tetramethyl-*p*-quaterphenyl (Exalite360), 3,5,3‴,5‴-tetra-*t*-butyl-*p*-quinquephenyl (QUI), 3,5,3‴″,5‴″-tetra-*t*-butyl-*p*-sexiphenyl (TBS) and [12] cycloparaphenylene (12-CPP).

Linear and recently reported cyclic oligo-*p*-phenyls with and without methyl and *t*-butyl substituents are among the simplest extended *π*-electron systems (Chart 4). Linear oligo-*p*-phenyls have been utilized as luminophores in dye lasers and scintillators in near-UV and visible region for many years [76,77,78] due to their photochemically and thermally robust features. Although unsubstituted oligo-*p*-phenyls longer than five or six repeating units have limited solubility, *p*-dioxane is commonly used as a solvent for reasons not fully understood by the authors. In contrast, methyl- and *t*-butyl-substituted oligo-*p*-phenyls show excellent solubility in common organic solvents. Nevertheless, unsubstituted cyclic-*p*-phenyls recently synthesized are also readily soluble in common organic solvents, permitting their utilization as luminophores in which the emission band is controllable by ring number [79,80]. Due to inherent repulsions in closely approaching Ar-*H*/*H*-Ar and/or Ar-CH_2_-H/*H*-Ar pairs, these linear and cyclic oligo-*p*-phenyls can twist in left or right directions cooperatively.

**Figure 4:**
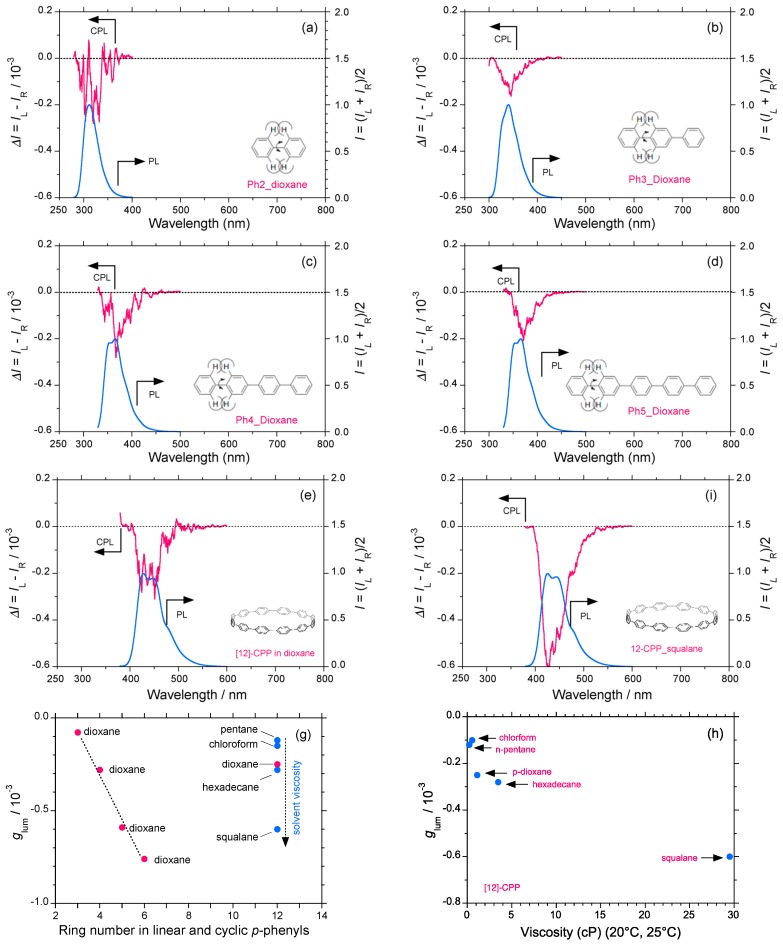
CPL/PL spectra of unsubstituted linear and cyclic oligo-*p*-phenyls in several solvents at room temperature (path length: 10 mm, cylindrical cuvette, conc.: (0.5–5) × 10^−5^ M: (**a**) CPL/PL spectra of Ph2 excited at 250 nm in; (**b**) CPL/PL spectra of Ph3 excited at 270 nm in *p*-dioxane; (**c**) CPL/PL spectra of Ph4 excited at 300 nm in *p*-dioxane; (**d**) CPL/PL spectra of Ph5 excited at 300 nm in *p*-dioxane; CPL/PL spectra of 12-CPP excited at 350 nm (**e**) in *p*-dioxane and (**f**) in squalene. Ph6 was insoluble or scarcely soluble in *p*-dioxane and other solvents; (**g**) the *g*_lum_ value at *λ*_ext_ of linear and cyclic oligo-*p*-phenyls in *p*-dioxane and other solvents as a function of phenyl ring number; (**h**) the *g*_lum_ value at *λ*_ext_ of 12-CPP in five solvents as a function of viscosity (in *cP*).

CPL/PL spectra of biphenyl (Ph2), *p*-terphenyl (Ph3), *p*-quaterphenyl (Ph4) and *p*-quinquephenyl (Ph5), in *p*-dioxane and [12] cyclo-*p*-phenylene (12-CPP) in p-dioxane and squalene are depicted in Figure 4a–f. Except for Ph2, CPL signals of Ph3, Ph4, Ph5 and 12-CPP in *p*-dioxane are obvious and their *g*_lum_ values range from −0.15 × 10^−3^ and −0.25 × 10^−3^. Although 12-CPP in the moderately viscous solvent *n*-hexadecane [3.47 *cP*] has weaker *g*_lum_ = −0.16 × 10^−3^ at 425 nm (Figure S3c in SM), 12-CPP in squalene (29.5 *cP*) is greatly increased to *g*_lum_ = −0.6 × 10^−3^ at 425 nm (Figure 4i) and CPL signals are obvious in chloroform, *n*-pentane and *n*-hexadecane (Figure S3a,b,d) in SM).

The *g*_lum_ value at *λ*_ext_ of Ph2, Ph3, Ph4, Ph5 and 12-CPP in *p*-dioxane and other solvents as a function of phenyl ring number is plotted in Figure 4g. Additionally, the *g*_lum_ value at *λ*_ext_ of 12-CPP as a function of the viscosity of the five solvents is plotted in Figure 4h. Evidently, the *g*_lum_ value of linear, non-rigid and oligo-*p*-phenyls in *p*-dioxane linearly amplifies to −0.8 × 10^−3^ when the phenyl ring number increases from 2 to 5, whilst that of semi-rigid 12-CPP decreases and is similar to the value for Ph4. Rotational freedom along C–C bonds between phenyl rings is likely to be crucial. Similarly, the *g*_lum_ value of 12-CPP amplified nonlinearly and leveled off at −0.6 × 10^−3^ as the viscosity increased. The viscosity of the solvents, number of phenyl rings and rotational freedom between phenyl rings appear to be crucial. However, the corresponding CD signals at the corresponding UV-vis spectra ofPh2, Ph3, Ph4, Ph5 and 12-CPP in *p*-dioxane are not obvious (Figure S3e–h in SM).

**Figure 5:**
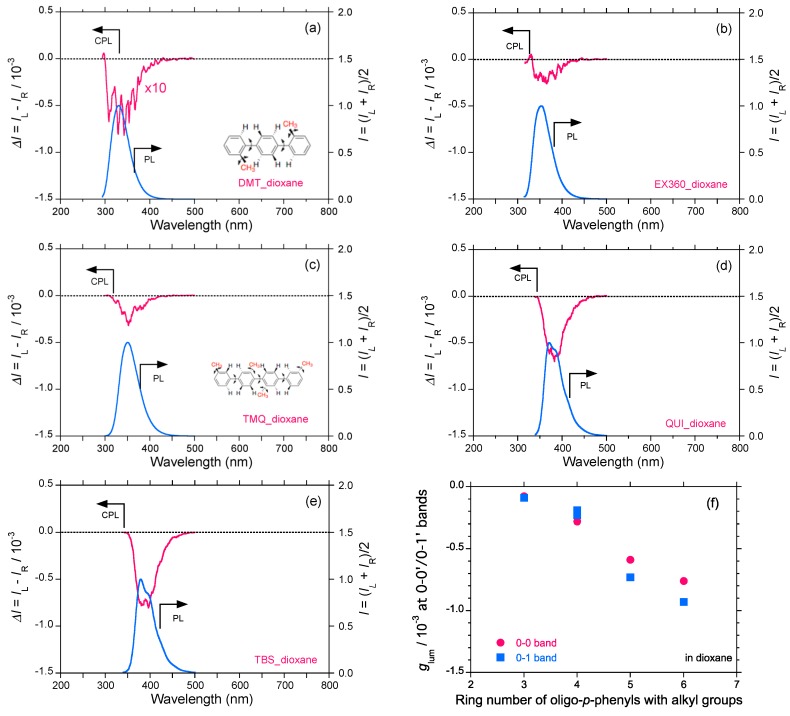
CPL/PL spectra of linear oligo-*p*-phenyls bearing methyl and *t*-butyl groups in *p*-dioxane at room temperature (path length: 10 mm, cylindrical cuvette, conc.: (2.5–10) × 10^−5^ M; CPL/PL spectra of (**a**) DMT excited at 265 nm, (**b**) Exalite360 excited at 285 nm, (**c**) TMQ excited at 270 nm, (**d**) QUI excited at 315 nm and (**e**) **TBS** excited at 315 nm; (**f**) the *g*_lum_ value at *λ*_ext_ of substituted oligo-*p*-phenyls in *p*-dioxane as a function of phenyl ring number in *p*-dioxane.

For comparison, we measured the CPL/PL spectra of highly soluble linear oligo-*p*-phenyl derivatives bearing methyl and *tert*-butyl side groups: 2,2″-dimethyl-1,1′:4′1″-terphenyl (DMT), 2,5,2‴,5‴-tetramethyl-*p*-quaterphenyl (EX360), 2″,3,3′,3‴-tetramethyl-1,1′:4′1″:4″,1‴-quaterphenyl (TMQ), 3,5,3‴′,5‴′-tetra-*t*-butyl-*p*-quinquephenyl (QUI) and 3,5,3‴″,5‴″-tetra-*t*-butyl-*p*-sexiphenyl (TBS) in *p*-dioxane (Figure 5a–e) and for comparison, their CD/UV-visible spectra in *p*-dioxane are displayed in Figure S4a–e in SM. DMT, EX360, TMQ, QUI and TBS in 1,4-dioxane, though, all exhibited distinct CPL signals at 333 nm, 352 nm, 350 nm, 372 nm and 380 nm, respectively, despite there being no detectable CD signals. The highest *g*_lum_ values for DMT, EX360, TMQ, QUI and TBS in *p*-dioxane were −0.08, −0.28, −0.74, −0.93 × 10^−3^ at the 0-0′ or 0-1′ band, respectively (Figure 5a–e). The highest *g*_lum_ value had a tendency to increase nonlinearly as phenyl ring number (*n*) increased from *n* = 3 to 6 (Figure 5f).

**Figure 6:**
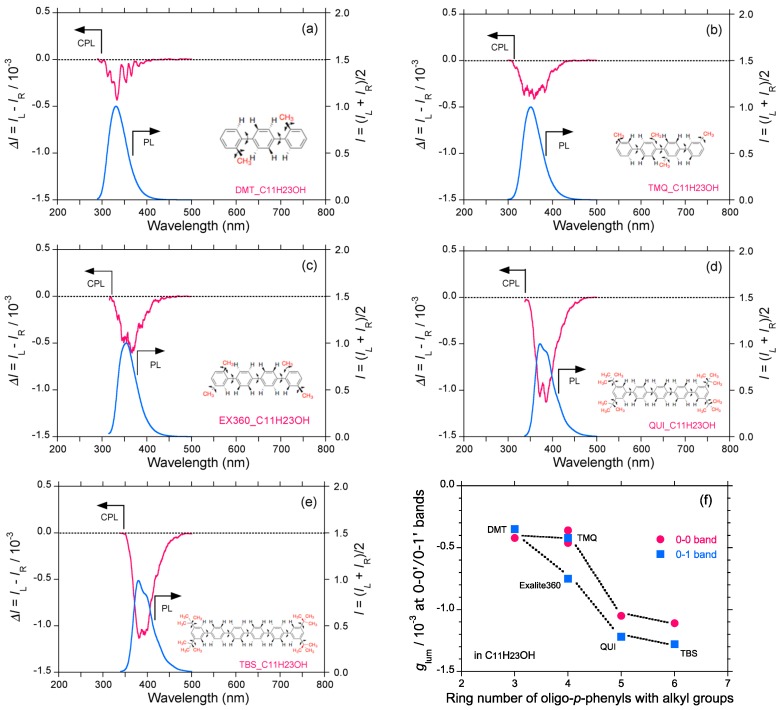
CPL/PL spectra of linear oligo-*p*-phenyls carrying methyl and *t*-butyl rotors in *n*-undecanol at room temperature (path length: 10 mm, cylindrical cuvette, conc.: (2.5–10) × 10^−5^ M: (**a**) CPL/PL spectra of DMT excited at 260 nm, (**b**) TMQ excited at 270 nml, (**c**) Exalite360 excited at 280 nm, (**d**) QUI excited at 310 nm and (**e**) TBS excited at 315 nm; (**f**) the *g*_lum_ values at the 0-0′ and 0-1′ bands of *λ*_ext_ of five oligo-*p*-phenyls with alkyl groups in *n*-undecanol as a function of phenyl ring number.

In the case of viscous *n*-undecanol, these *g*_lum_ values increased to DMT −0.42 × 10^−3^ at the 0-0′ and 0-1′ bands (Figure 6a), Exalite 360 −0.75 × 10^−3^ at the 0-1′ band (Figure 6b), TMQ −0.42 × 10^−3^ at the 0-1′ band (Figure 6c), QUI −1.22 × 10^−3^ at the 0-1′ band (Figure 6d) and TBS −1.28 × 10^−3^ at the 0-1′ band (Figure 6e). The highest *g*_lum_ value in *n*-undecanol had a tendency to increase nonlinearly as the phenyl ring number (*n*) increased from *n* = 3 to 6 (Figure 6f). Obviously, the *g*_lum_ values of QUI and Ex360 in *n*-undecanol at 0-1′ band are stronger than those at the 0-0′ band, due to the Franck-Condon principle and the great difference in potential energy surfaces and nuclear coordinates between the *S*_1_ and *S*_0_ states. Methyl and *tert*-butyl groups attached to the *π*-conjugated framework efficiently increased photoexcited state chirality. Efficient coupling between the electronic structure of the *π*-electron system and the vibration modes of the *π*-conjugated framework at the *S*_1_ state may be crucial. The highest *g*_lum_ value at the 0-1′ band in *n*-undecanol tended to increase nonlinearly as the number of Ph rings increased from *n* = 3 to 6, reaching a plateau value of −1.3 × 10^−3^ (Figure 6f). Negative-sign CPL signals of EX360 and TBS in viscous ethylene glycol, 1,3-propanediol and 1,4-butanediol and even in *n*-pentane are obvious (Figure S4f–j in SM).

### 2.5. Chain-Like Non-Rigid Oligofluorene Derivatives Bearing Alkyl Groups

**Chart 5:**
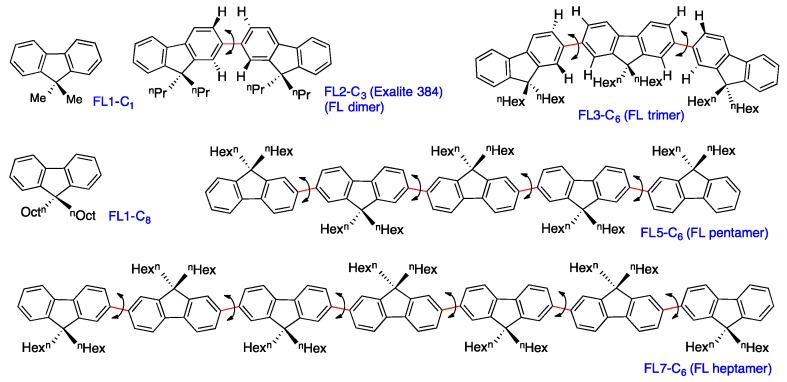
Chemical structures of 9,9-dimethylfluorene (FL1-C_1_), 9,9-di-*n*-octyllfluorene (FL1-C_8_), 9,9,9′,9′-tetrakis(*n*-propyl)-2,7′-difluorene (Exalite 384), 9,9,9′,9′,9″,9″-hexakis(*n*-hexyl)-2,7′;2′,7″- trifluorene (FL3-C_6_), 9,9,9′,9′,9″,9″,9‴,9‴,9″″,9″″-decakis(*n*-hexyl)-2,7′;2′,7″,2″,7‴;2‴,7″″-pentafluorene (FL5-C_6_), 9,9,9′,9′,9″,9″,9‴,9‴,9‴′,9‴′,9‴″,9‴″,9‴‴,9‴‴-dodecakis(*n*-hexyl)-2,7′;2′,7″,2″,7‴;2‴,7‴′,2‴′, 7‴″, 2‴″,7‴‴-heptafluorene (FL7-C_6_).

Oligo- and polyfluorenes bearing alkyl chains in the 9,9-positions of the fluorene ring are known to be excellent emitters with a high PLQY of greater than 0.9 at ambient temperature, even in solution [81,82,83]. The *C*_2v_-symmetrical fluorene ring has V-shaped topology in a planar geometry and can be regarded as a twistable biphenyl derivative (Ph2). When two fluorene rings connect to each other at the 2- and 7-positions, even the simplest fluorene dimer adopts four possible geometries: polar *C*_2_-symmetrical syn-forms with *P*- and *M*-helix senses and less-polar *C*_1_-symmetrical anti-forms with *P*- and *M*-helix senses. With extension of the fluorene repeat unit (*N*), the possible conformations (rotamers) increase non-linearly, obeying a 4^(*N*−1)^ function due to the rotational freedom between the fluorene rings. A series of 9,9-dialkylfluorene oligomers (*N* = 1,2,3,5,7) (Chart 5) dissolved in various alkanes, alcohols and other solvents permits discussion of CPL/PL and CD/UV-visible spectral characteristics as functions of *N* and solvent viscosity based on knowledge of Section 2.2, Section 2.3, Section 2.4 and Section 2.5.

**Figure 7:**
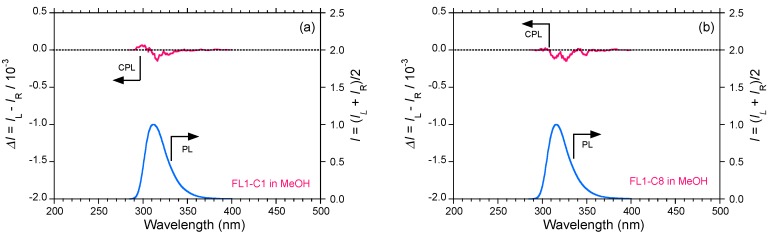
CPL/PL spectra of (**a**) FL1-C_1_ excited at 255 nm and (**b**) FL1-C_8_ excited at 255 nm at room temperature in methanol. Path length: 10 mm, cylindrical cuvette, conc.: 5 × 10^−5^ M.

First of all, the CPL/PL spectra of 9,9-dimethylfluorene (FL1-C_1_) and 9,9-dioctylfluorene (FL1-C_8_) in methanol and a higher viscous *n*-undecanol (16.95 *cP*) are compared in Figure 7a,b. Although CD Cotton effects at the corresponding UV bands for FL1-C_1_ and FL1-C_8_ in methanol are not detectable (Figure S5a,b) in SM), very weak CPL signals are nevertheless apparent; a bisignate CPL band for FL1-C_1_ in methanol can be seen, although for FL1-C_1_ in *n*-undecanol and FL1-C_8_ in methanol and *n*-undecanol, weak (–)-sign CPL bands are reproducibly observed (Figure 7a,b and Figure S5c,d) in SM). These FL monomers at the S_1_ state may be slightly distorted because of two alkyl groups located at the same 9,9-positions. These *g*_lum_ values, on the order of 10^−4^ and with a (–)-sign, are negligibly small when compared to those of oligofluorenes (*N* = 2–7), as shown in the flowing.

**Figure 8:**
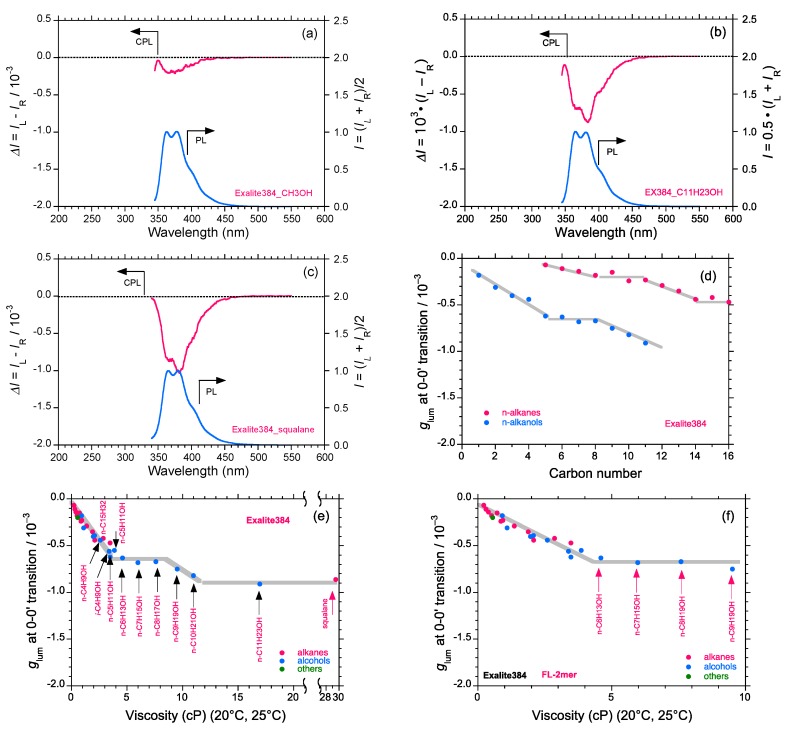
CPL/PL spectra of FL2-C_3_ (Exalite 384, fluorene dimer) excited at 315 nm at room temperature (**a**) in methanol, (**b**) in *n*-undecanol and (**c**) in squalene; (**d**) the *g*_lum_ value at 0-0’ band of FL2-C_3_ as a function of carbon numbers in two series of *n*-alkanes and *n*-alkanol including methanol and ethanol; (**e**) the *g*_lum_ value at the 0-0’ band of FL2-C_3_ (Exalite 384) as a function of viscosity (in *cP* at 20–25 °C) in various achiral alkanes, alkanols and other solvents and (**f**) magnification of the viscosity region 0–10 cP. Path length: 10 mm, cylindrical cuvette, conc.: 1 × 10^−5^ M.

Next, the CPL/PL spectra of Exalite 384 (a fluorene dimer, FL2-C_3_), bearing *n*-propyl groups in the 9,9′-positions, in several solvents were compared (Figure 8a–c and Figure S6a–c) in SM), including for reference the CD/UV-visible spectra in methanol (Figure S6d in SM). The fluorene dimer clearly showed (–)-sign vibronic CPL signals at 360–380 nm. In lower viscosity solvents such as methanol, *n*-pentane and chloroform (0.55 *cP*), the *g*_lum_ values are of the order of −(0.05~0.2) × 10^−3^ (Figure 8a and Figure S6a,c in SM). However, in *n*-hexadecane (3.47 *cP*), *n*-undecanol (16.95 *cP*) and the branched hydrocarbon, squalene (29.5 *cP*), the *g*_lum_ values increase to −0.5 × 10^−3^, −0.9 × 10^−3^ and −1.1 × 10^−3^, respectively (Figure 8b,c and Figure S6b in SM). The *g*_lum_ value was plotted as carbon numbers of *n*-alkanes (*N* = 5–16) and *n*-alkanols including methanol and ethanol (*N* = 1–11) in Figure 8d. It is obvious that the *g*_lum_ value increases discontinuously with increase of the carbon numbers in the two series of *n*-alkanes and *n*-alkanols. Larger carbon numbers increase the viscosity due to an increase in London dispersion interactions and energy. 

This tendency led us to plot the *g*_lum_ value as a function of solvent viscosity using thirty-two kinds of molecular liquids, including twelve alkanes (linear and branched), eighteen alcohols (linear, branched and cyclic) and chloroform (Figure 8e,f)). The *g*_lum_ value increased but underwent a discontinuous step-like transition when [*η*] = 4–9 *cP* and 11–30 *cP* (Figure 8e). The *g*_lum_ value increased almost linearly in response to solvent viscosity when [*η*] was in the range 0.21 (*n*-pentane) to [*η*] = 4.59 (*n*-hexanol) (Figure 8e,f).

The quantized, two-step-like phase transition behaviors should be closely related to structural reorganization at the S_1_ state. A possible explanation for the quantized *g*_lum_–[*η*] relation arises from anti-syn structural reorganization occurring at the S_1_ state associated with a change in preference in twisted geometry between left and right. Exalite 384 in solution at the S_0_ state should exist as a mixture of two enantiomeric pairs of anti and syn forms, as proven by the lack of CD signals at 320 nm for the π-π* transition of the fluorene rings. The less polar anti-form is energetically more stable than the polar *syn* form. However, Exalite 384 at the S_1_ state prefers the polar syn-form with a handed twist due to suppression of ro-vibrational modes along the single C–C bond between the two fluorene rings when [*η*] is sufficient high. At the first transition when [*η*] = 5–7 *cP*, Exalite 384 adopts the anti-form with a *P*-screw sense (dihedral angle between fluorene ring +40°) exhibiting (–)-sign CPL signals. At the second transition when [*η*] > 11 *cP*, Exalite 384 adopts the more stable *syn* form with an *M*-screw sense (dihedral angle between fluorene ring −40°), exhibiting (−)-sign CPL signals. 

The *g*_lum_–[*η*] relation of the fluorene dimer bearing *n*-propyl side chains (Exalite 384) prompted us to further investigate the fluorene-ring number (*N*) dependence of the CPL/PL and CD/UV-visible spectral characteristics in three fluorene oligomers bearing *n*-hexyl groups, including a fluorene trimer (FL3-C_6_), a fluorene pentamer (FL5-C_6_) and a fluorene heptamer (FL7-C_6_).

**Figure 9:**
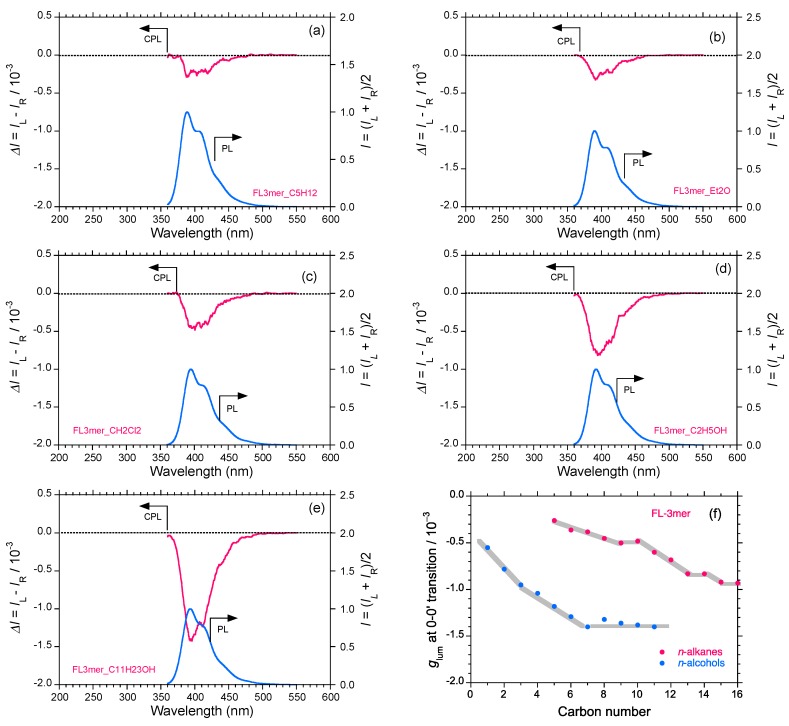
CPL/PL spectra of FL3-C_6_ (fluorene trimer) excited at 330 nm at room temperature (**a**) in *n*-pentane, (**b**) in diethylether, (**c**) in dichloromethane, (**d**) in ethanol and (**e**) in *n*-undecanol; (**f**) The *g*_lum_ value at 0-0′ band of FL3-C**_6_** as a function of carbon numbers in two series of *n*-alkanes and *n*-alkanol (including ethanol); (**g**) the *g*_lum_ value at the 0-0’ band of FL3-C_6_ as a function of viscosity (in *cP* at 20–25 °C) of various achiral alkanes, alkanols and other solvents and (**h**) magnification of the viscosity region 0–2.0 cP. Path length: 10 mm, cylindrical cuvette, conc.: 2 × 10^−5^ M.

The CPL/PL spectra of FL3-C_6_ are displayed in Figure 9a–e and Figure S7a–c in SM and for comparison CD/UV-visible spectra in ethanol are given in Figure S7d in SM. FL3-C_6_ reveals clear CPL signals at the same wavelength as the PL in the emission spectra in low viscosity solvents like *n*-pentane (0.21 *cP*, Figure 9a), diethylether (0.22 *cP*, Figure 9b), dichloromethane (0.41 *cP*, Figure 9c), ethanol (1.09 *cP*, Figure 9d), acetone (0.31 *cP*, Figure S7a in SM), tetrahydrofuran (0.46 *cP*, Figure S7b in SM) and can also be observed in higher viscosity solvents including *n*-undecanol (16.95 *cP*, Figure 9e) and squalene (29.5 *cP*, Figure S9c in SM). By testing with twelve *n*-alkanes, fourteen alkanols (linear and branched) and seven other types of solvents (diethylether, THF, chloroform, dichloromethane, acetone, isooctane and squalene), the full-range *g*_lum_–[η] and its zoomed-in *g*_lum_–[*η*] relations were evaluated and are plotted in Figure 9g,h, respectively. The *g*_lum_ value as a function of carbon numbers of *n*-alkanes and *n*-alkanols including ethanol indicated step-like transitions at specific carbon numbers, indicating that the effect is a function of [*η*] value and does not depend on the chemical identity of the alkane or alkanol (Figure 9f). The *g*_lum_ values tended to alter linearly in two steps when [*η*] is in the ranges between 0.2 to 1.0 *cP* and from 1 to 6 *cP*. Eventually, the *g*_lum_ value leveled off at −1.40 × 10^−3^ at 390 nm when [*η*] > 7 *cP*.

**Figure 10:**
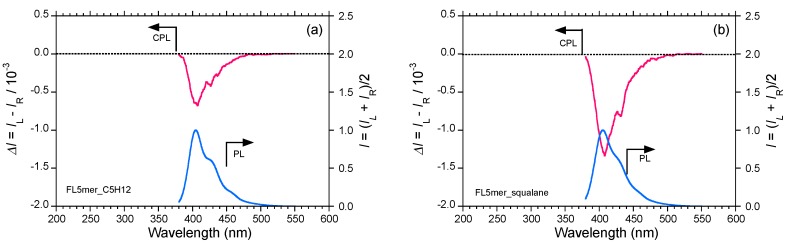

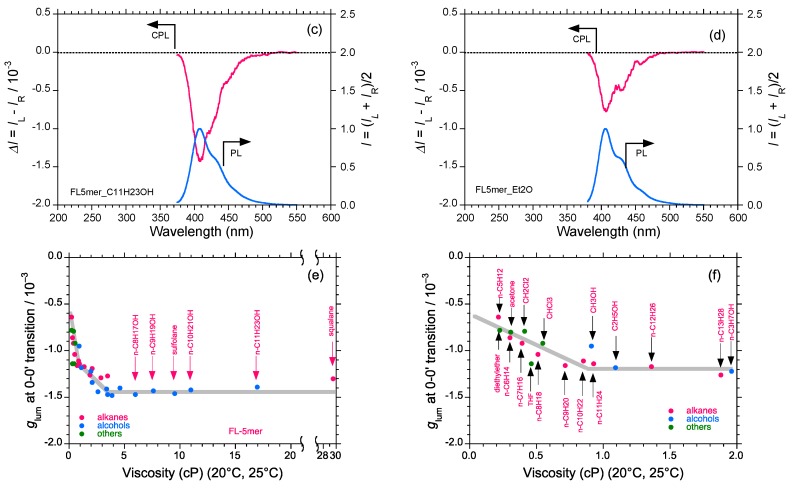
CPL/PL spectra of FL5-C_6_ (fluorene pentamer) excited at 340 nm at room temperature (**a**) in *n*-pentane, (**b**) in squalene, (**c**) in *n*-undecanol and (**d**) in diethylether; (**e**) the *g*_lum_ value at the 0-0′ band of FL5-C_6_ as a function of viscosity (in *cP* at 20–25 °C) of various achiral alkanes, alkanols and other solvents and (**f**) magnification of the viscosity region 0–2.0 cP. Path length: 10 mm, cylindrical cuvette, conc.: 5 × 10^−6^ M.

Similar tendencies in the CPL/PL/CD/UV-visible spectral characteristics of FL5-C_6_ can be seen in several solvents of lower and higher viscosity (Figure 10a–d and Figure S8a–b) in SM). FL5-C_6_ revealed significant CPL signals at the corresponding PL emissions, regardless of solvent viscosity. By using twelve *n*-alkanes, fourteen alkanols (linear and branched) and six other types of solvents (diethylether, THF, chloroform, dichloromethane, acetone and squalene), the full-range *g*_lum_–[*η*] and its zoomed-in *g*_lum_–[*η*] relations were evaluated and are plotted in Figure 10e,f, respectively. The *g*_lum_ value as a function of carbon numbers of *n*-alkanols including ethanol indicated a clear transition at a carbon number of four (Figure S8d in SM). From Figure 10e–f, the *g*_lum_ values tended to alter linearly in two steps when the [*η*] value was in the ranges 0.2 *cP* to 0.7 *cP* and 0.7 *cP* to 4 *cP*. Eventually, the *g*_lum_ value leveled off at −1.44 × 10^−3^ at 407 nm when [*η*] > 4 *cP*. 

**Figure 11:**
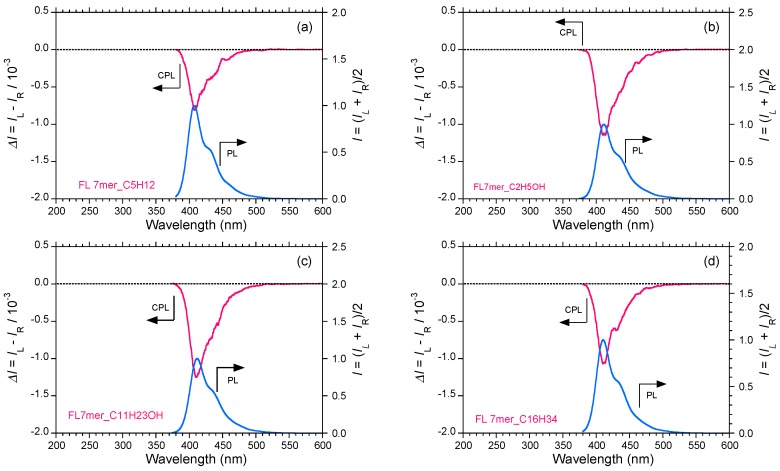

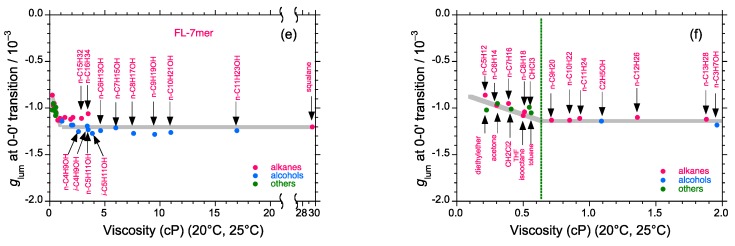
CPL/PL spectra of FL7-C_6_ (fluorene heptamer) excited at 350 nm at room temperature (**a**) in *n*-pentane, (**b**) in ethanol, (**c**) *n*-undecanol and (**d**) in *n*-hexadecane; (**e**) the *g*_lum_ value at the 0-0′ band of FL7-C_6_ as a function of viscosity (in *cP* at 20–25 °C) of various achiral alkanes, alkanols and other solvents and (**f**) magnification of the viscosity region 0–2.0 cP. Path length: 10 mm, cylindrical cuvette, conc.: 5 × 10^−6^ M.

Very similar CPL/PL/CD/UV-visible spectral characteristics for FL7-C_6_ were also confirmed (Figure 11a–d and Figure S9a–d in SM). For comparison, CD/UV-visible spectra in ethanol are given in Figure S9e in SM. FL7-C_6_ revealed clear CPL signals at the corresponding to PL spectra in low viscosity solvents (*n*-pentane, diethylether, dichloromethane, acetone and chloroform) and in viscous solvents like *n*-undecanol and *n*-hexadecane. By using the same series of *n*-alkanes and alkanols and other types of solvents used for FL5-C_6_, the full-range *g*_lum_–[*η*] and its zoomed-in *g*_lum_–[*η*] relations were evaluated and are plotted in Figure 11e,f, respectively. The *g*_lum_ value as a function of carbon numbers of *n*-alkanes and *n*-alkanols including ethanol indicated transitions when the carbon numbers in the *n*-alkanes was four and in *n*-alkanols was six and nine (Figure S9f in SM). The *g*_lum_ values alter linearly in one-step only when [*η*] is in the range from 0.2 *cP* to 0.6 *cP*. Eventually, the *g*_lum_ value leveled off at −1.25 × 10^−3^ at 412 nm when [*η*] > 0.6 *cP*.

**Chart 6:**
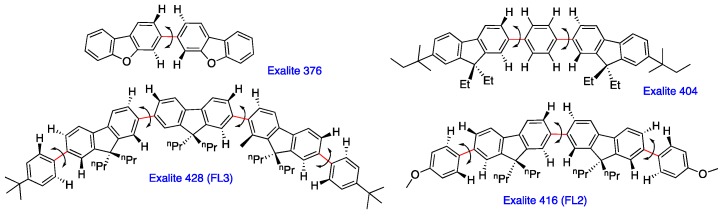
Chemical structures of 3,3′-bidibenzo[b,d]furan (Exalite 376), 1,4-bis(9,9-diethyl-7-(*t*- pentyl)-9H-fluoren-2-yl)benzene (Exalite 404), 7,7″-bis(4-(*t*-butyl)phenyl)-1-methyl-9,9,9′,9′,9″,9″- hexapropyl-9H,9′H,9″H-2,2′:7′,2″-terfluorene (Exalite 428) and 7,7′-bis(4-methoxyphenyl)-9,9,9′,9′- tetrapropyl-9H,9′H-2,2′-bifluorene (Exalite 416).

**Figure 12:**
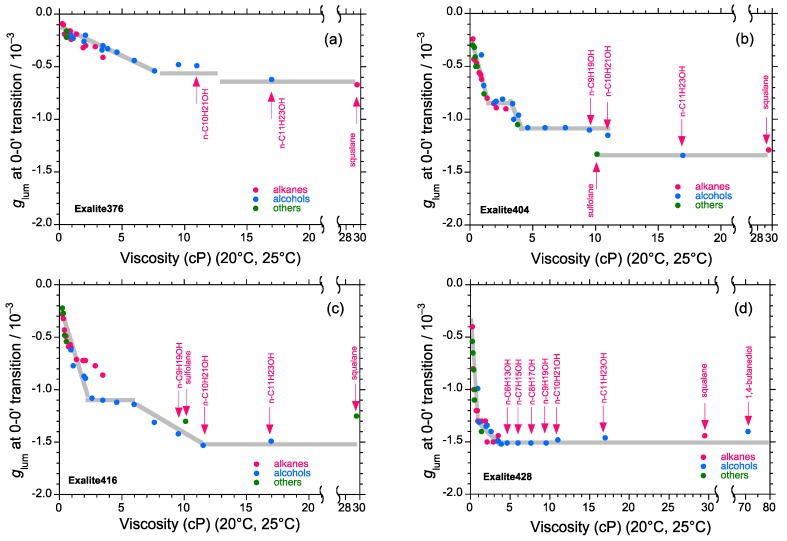

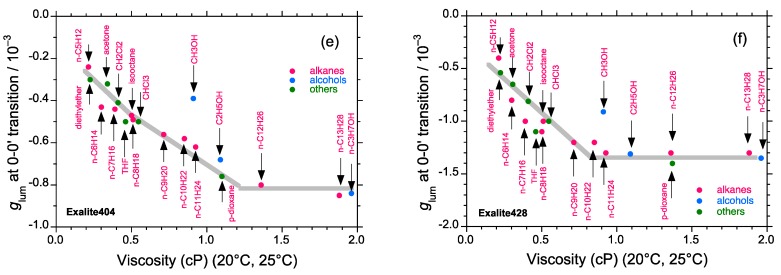
The *g*_lum_ value at th 0-0′ band of (**a)** Exalite 376, (**b**) Exalite 404, (**c**) Exalite 416 and (**d**) Exalite 428 as a function of the viscosity (in *cP* at 20–25 °C) of various achiral alkanes, alkanols and other solvents (0–30 cP). The magnified plots of *g*_lum_ value at 0-0′ band of (**e**) Exalite 404 and (**f**) Exalite 428 as a function of the viscosity (in *cP* at 20–25 °C) of various achiral alkanes, alkanols and other solvents (0–2.0 cP). Path length: 10 mm, cylindrical cuvette, conc.: 1 × 10^−5^ M.

Four other commercially available fluorene analogs, Exalite 376 (dibenzofuran dimer), Exalite 404 (*p*-phenylene connected to two fluorenes), Exalite 428 (fluorene trimer bearing two *p*-*tert*-butylphenyl groups) and Exalite 416 (fluorene dimer bearing two *p-*anisyl groups) (Chart 6), showed similar *g*_lum_–[*η*] tendencies over a broad range of [*η*] values (Figure 12a–f). Exalite 376 and Exalite 384 are in the same category because they have one rotatable C–C single bond between the two fluorenes or two dibenzofurans. Similarly, Exalite 404, Exalite 384, Exalite 416 and FL3-C_6_ are in the same category because they have two rotatable C–C single bonds between three fluorenes (and *p*-phenylene), if the very floppy end-termini of the two aryl groups are ignored. With further increase in the fluorene ring number from three to five and seven, the absolute *g*_lum_ value tended to decrease slightly.

**Figure 13:**
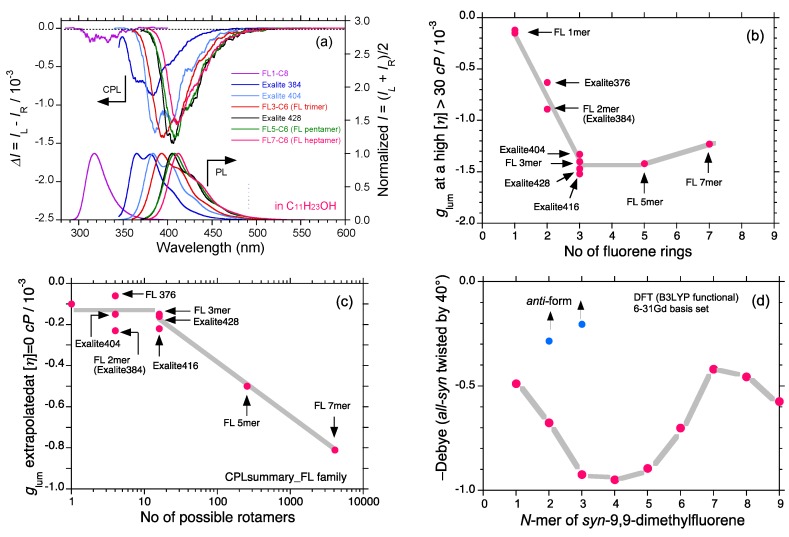

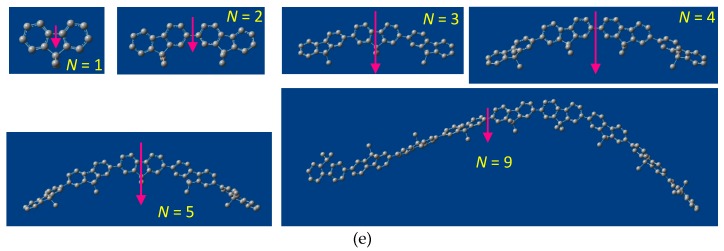
(**a**) Comparisons of CPL and PL spectra at the 0-0′ band of FL1-C_8_, Exalite 384, Exalite 404, FL3-C_6_, Exalite 428, FL5-C_6_ and FL7-C_6_ in *n*-undecanol; (**b**) the *g*_lum_ value at the 0-0′ band extrapolated at [*η*] > 30 cP of FL1-C_8_, Exalite 384, Exalite 404, FL3-C_6_, Exalite 428, FL5-C_6_ and FL7-C_6_ as a function of the fluorene ring number; (**c**) the *g*_lum_ value at the 0-0’ band extrapolated at [*η*] = 0.0 **cP** of **FL1-C_8_,** Exalite 384, Exalite 404, FL3-C_6_, Exalite 428, FL5-C_6_ and FL7-C_6_ as a function of possible rotational isomers of these fluorenes; (**d**) the dipole moments (in Debye) of hypothetical 9,9-dimethylfluorene *N*-mers, adopting a syn-geometry twisted by 40° in a vacuum as a function of repeat number *N*. For comparison, the dipole moments (in Debye) of hypothetical 9,9-dimethylfluorene dimer and trimer, adopting an anti-geometry twisted by 40° in a vacuum were marked in blue dots. These dipole moments were obtained using DFT (B3LYP with 6-31G(d) basis set); (**e**) optimized models of fluorene *N*-mer (*N* = 1–5 and 9), the molecular models of the 9,9-dimethylfluorene *N*-mer with *N* = 1, 2, 3, 4, 5 and 9, optimized by DFT. The red arrow stands for the relative magnitude of the dipole moments. As *N* increases from 1 to 5, the dipole moment increases monotonically, while when *N* = 9, the dipole moment abruptly decreases due to a partial cancellation of the moments in the case of a helical geometry that is uniformly twisted by 40° macroscopically.

To this end in the study of the chiroptical characteristics in the fluorene family at the *S*_1_ state, we compared the CPL and PL spectra of FL1-C_8_, Exalite 384, Exalite 404, Exalite 428, FL3-C_6_, FL3-C_6_, FL5-C_6_ and FL7-C_6_ dissolved in *n*-undecanol (16.95 *cP*) in Figure 13a. The *λ*_ext_ peak wavelength of the vibronic luminescence bands at the 0-0′ transition progressively redshifted as the fluorene ring number increased from one to seven. The magnitude of the CPL signal, *∆I* = *I*_L_ − *I*_R_, increased when the ring number was greater than three (regarding Exalite 404 as a fluorene trimer).

From the empirical *g*_lum_–[*η*] relations of Exalite 384, FL3-C_6_, FL5-C_6_, FL7-C_6_, Exalite 376, Exalite 404, Exalite 428 and Exalite 416 (Figure 8e–h, Figure 10e,f, Figure 11e,f and Figure 12a–f), it becomes apparent that the [*η*] value may be extrapolated to a non-zero *g*_lum_ value with (–)-sign when [*η*] is 0.0 *cP*, corresponding to solvent-free, collision-free conditions. This tendency is remarkable in the cases of FL5-C_6_ (Figure 10f) and FL7-C_6_ (Figure 11f). For *g*_lum_ values at [*η*] > 30 *cP*, this tendency is obvious as a function of fluorene ring number (Figure 13b). It is clear that the absolute *g*_lum_ value increases when *N* = 1–3 and levels off at *N* = 5 and decreasing slightly when *N* = 7.

To explain this tendency, we assumed that photoexcited fluorene *N*-mers in the S_1_ state prefer a polar geometry, adopting the syn-form with +40° twists. Molecular models of the 9,9-dimethylfluorene *N*-mers (*N* = 1, 2, 3, 4, 5 and 9) optimized by DFT calculation (B3LYP functional with 6-31G(d) basis set) are illustrated in Figure 13e. The red arrow relates to the relative magnitude of the dipole moments. As the *N*-number increases from 1 to 3, the dipole moment increases monotonically. However, the magnitude of the dipole moments leveled off at *N* = 3–5 and then decreased for *N* = 5–7 due to the mutual cancellation between all the moments of the macroscopically twisted helical geometry (Figure 13e, *N* = 9). For comparison, the fluorene *N*-mers (*N* = 2, 3) with anti-form twisted by +40° are less polar and more likely to depend weakly on *N* (blue filled circles, Figure 13e). Possibly fluorene *N*-mers (*N* ≥ 2) in the S_1_ state may adopt a similar twisted syn-form rather than a twisted anti-form.

The numbers of rotamers in these compounds, arising due to the degree of freedom, is connected to cosmological entropy in the Big Bang Universe [84,85,86]. A larger number of rotamers means a high entropy state and conversely, one rotamer indicates zero entropy. The rotational barrier (*E*_b_) in oligo- and polyfluorenes is as small as ~0.6 kcal mol^−1^ per C–C single bond between the rings [87]. This small *E*_b_ value allows for generation of all possible rotamers in a statistical fashion under thermodynamic equilibrium since the thermal energy at 300 K is ~0.6 kcal mol^−1^. Although **Exalite 384** with one rotatable C–C bond has four possible rotamers (syn- and anti-) with *P*- and *M*-twists as mentioned above, FL7-C_6_ can in principle adopt 4096 rotamers as it has six rotatable C–C bonds. Thus, **FL7-C_6_** in the highest entropy *S*_0_ state reveals the greatest (–)-sign *g*_lum_ value arising from temporal generation of a few rotamers on the order of ~10^−8^ s obeying Kasha’s rule.

We re-plotted the *g*_lum_ value extrapolated at [*η*] = 0 *cP* as a function of the number of possible rotamers in a family of nine fluorenes on a logarithmic scale (Figure 13c). Although the *g*_lum_ value at zero-viscosity shows a constant value of ~ −0.2 × 10^−3^, up to possible rotamers (~15), the value increases exponentially and levels off at ~ −0.8 × 10^−3^ as the number of possible rotamers increases. The *g*_lum_ value at zero-viscosity implies that the photoexcited oligofluorenes will emit CPL signals with (–)-sign even under collision-free conditions, such as, for example, in a vacuum.

## 3. Discussion

One can imagine that the huge numbers of the far off fixed stars in the accelerating expanded Universe, excepting our solar sun, are faintly shining as a result of proton-proton chain reactions between hydrogen atoms that are producing deuterium, positrons and neutrinos, induced by the *PV* weak nuclear force mediated by the charged *W*^+^ boson [88]. Due to a high Coulombic barrier height between repulsive protons, the reactions occur only very slowly by a tunneling mechanism [88]. Due to the intensity of the light from our sun, and its subsequent scattering in the earth’s atmosphere by dust, water mist and other materials, the faintly shining stars are not apparent at all during the daytime. However, people can enjoy the shining only at nighttime when the influence of the sun is absent. 

Similarly, if one wishes to detect the weak shining chiroptical signals coming from the inherent left-right imbalance between enantiomers due to the *PV*-weak nuclear force originating from the neutral *Z*^0^ boson, it is necesssary to remove completely any influences of the parity-conserving *EM* force between enantiomers, that already adopt chirally distorted rigid frameworks due to the existence of point chirality and/or atrope chirality. A CPL spectropolarimeter which measures steady state systems may be regarded as a ‘*desk-top low-energy spinning photon–molecule collider decelerator*’ as it allows for the detection of the subtle difference in left- and right-handed light speed between enantiomers at the *S*_1_ state, which are temporally generated with lifetimes on the order of several 10^−9^ s during irradiation of an unpolarised photon source, while a CD spectropolarimeter, operating on the timescale of 10^−15^ s and based on the Franck-Condon principle is the low-energy photon–molecule interaction analyzer to detect differences between thermally stable enantiomers with long lifetimes at the *S*_0_ state.

The CPL spectrometer can excite huge numbers of molecular luminophores at once. In the relaxation process from the *S*_1_ to *S*_0_ state in a cylindrical cuvette (path length 1.0 cm, 10^−6^–10^−5^ M) with front-side lens-focused beam (~1 mm in diameter), approximately, 10^13^–10^14^ molecules are excited simultaneously and macroscopically. The relaxation process obeys the *arrow of time* proposed by British astronomer, Arthur Eddington in 1927 [89]; this concept means that entropy increases spontaneously as time goes by. The arrow of time implies the asymmetry of time that discriminates between future, present, and past. This direction is pre-determined by organization of subatoms, atoms, molecules, polymers and life in the framework of the four-dimensional relativistic world. If it is possible to extend the concept to the issue of spontaneous and/or mirror symmetry- breaking (MSB) and *MPV* hypotheses, the left-right direction is pre-determined by the *PV-WNC*, which is mediated by a handed *Z*_0_ boson and for non-rigid photoexcited molecules result in a preference to radiate right-handed circularly polarized light.

When the molecules are excited by photon sources, the PMT detector of the CPL spectrometer (present) can detect the passage of radiation (past) at the *S*_1_ state, an event which occurred 10^−9^ s before, whilst the PMT detector of the CD spectrometer (present) can see the passage of absorption at the *S*_0_ state which occurred 10^−15^ s before (past) based on the Franck–Condon principle and the Kasha’s rule in the Jablonski diagram [66,67,90].

In the past, our Universe experienced in the past three major phase transitions as a consequence of three broken symmetries to produce four fundamental physical forces that govern our life and matter worlds [91,92,93,94]. The timeline from the Big Bang (BB) to present is considered as follows: 

The four forces were unified in the beginning of the Universe, at the so-called Planck time (~10^−44^ s after BB). Then firstly, the unified force bifurcated into gravitational and strong-and-electroweak forces at ~10^−42^ s, followed by bifurcation of the strong-and-electroweak force into strong and electroweak forces at ~10^−34^ s. Eventually, at ~10^−12^ s, the electroweak force bifurcated into the electromagnetic and weak forces, producing unequal bosons (massive W^±^ and Z^0^ and the massless photon). This event occurred in a period of *ca*. 10^−15^ s, and as an event shorter than 10^−12^ s governed by the electroweak force, may maintain mirror symmetry. However, for events occurring over a period of 10^−9^ s, i.e., longer than 10^−12^ s, it is possible to break the mirror symmetry governed by the weak force. Thus, an event timescale of ~10^−12^ s may be critical in the question of whether one can detect mirror symmetry conservation or mirror symmetry breaking spectroscopically.

As mentioned in the Introduction, Hund showed a theoretical relationship between molecular chirality and optical activity for a hypothetical chiral molecule in a mirror symmetric DW [1,2]. Based on the idea of time-dependent, non-stationary states of a hypothetical chiral molecule in the DW and quantum tunneling, the theory showed a solution in which the tunneling causes energy splitting, *∆E*_±_, in the DW, leading to a spontaneous oscillation in chirality and optical activity with a dynamic racemization time (*T*_rac_) [1,2,3,34].

*∆E*_±_ = 1/2⋅π^−1/2^⋅(*hυE*_b_)^1/2^⋅exp(*–E*_b_/*hυ*) (1)

where *E*_b_ is the height of the energy barrier at the *S*_o_ state and is possibly also applicable to the *S*_1_ state, *υ* is the frequency for the vibration in a single-well, *h* is Planck’s constant. 

Also, the relationship between energy splitting and racemization time [1,2] may be given as

*T*_rac_ = *h*/*∆E*_±_ (2)

From Equation (1), the tunneling-dependent racemization time is predominantly determined by the value *of E*_b_, and has no temperature (*T*) dependency. This situation is very different from that of the thermally activated racemization process expressed by the Arrehenius equation, *k* = *A*⋅exp(*–E*_B_/(*N*⋅*k_B_*⋅*T*)), where *k* is the rate constant, *k_B_* is the Boltzmann constant, *N* is Avogadro’s number*,* and *A* is the collision factor. A demarcation between the tunneling and Arrehenius-type racemizations will be that the tunneling occurs when *E*_b_/*Nk_B_T* < 10 (~6 kcal mol^−1^) and the thermally racemization is a dominant process when *E*_b_/*Nk_B_T* > 30 (~20 kcal mol^−1^). Actually, the *E*_b_ value for the inversion of of ammonia is 5.8 kcal mol^−1^ [1,4]. Stochastic behavior is expected, when 10 < *E*_b_/*Nk_B_T* < 30. The idea of the *E*_b_/*Nk_B_T* relation might be applicable to the metastable *S*_1_ state.

**Figure 14:**
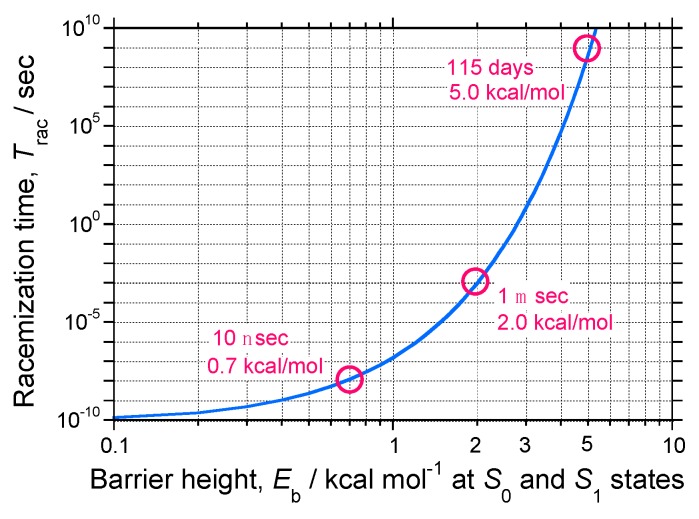
Plots of *E*_b_-dependent *T*_rac_ relationship, while *T*_rac_ = 10^−10^⋅sqrt(pi)/sqrt(c0⋅111) exp(c0/0.110): c0 = 0.1–10.0 with 0.10 interval.

To view schematically the *E*_b_-*T*_rac_ relationship, *T*_rac_ is illustrated (in s) as a function of *E*_b_ (in kcal mol^−1^) in *log*-*log* plots as shown in Figure 14. For simplicity, we chose one adjustable parameter, c0 = 0.10…10.0 with intervals of 0.10; the constant is 10^−10^⋅√*π* and *h*ν is fixed at 0.110. The value of *T*_rac_, that is the lifetime of one enantiomeric species, is 10^−8^ s, the representative lifetime of fluorescence, when *E*_b_ is 0.7 kcal mol^−1^. The *T*_rac_ value becomes 10^−3^ s longer, the representative lifetime of phosphorescence, when *E*_b_ slightly increases to 2.0 kcal mol^−1^. Moreover, the *T*_rac_ value becomes 115 days, the representative long lifetime of stable chirality, when *E*_b_ increases to 5.0 kcal mol^−1^. Under these conditions, stable optical activity at the *S*_0_ and *S*_1_ states is observable as CD and CPL signals. The next requirement is how the condition of *E*_pv_ ~ *E*_±_ is realized.

**Figure 15:**
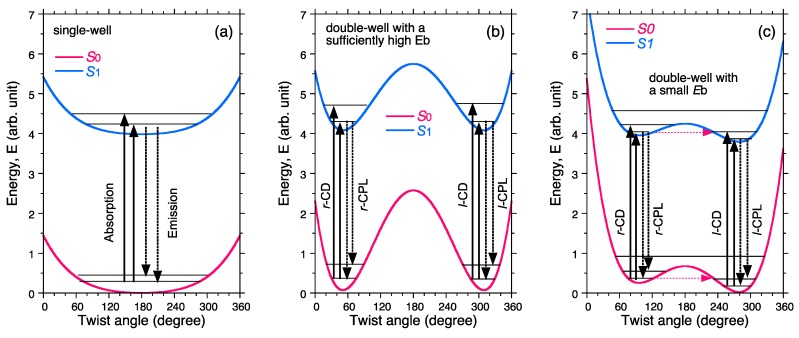

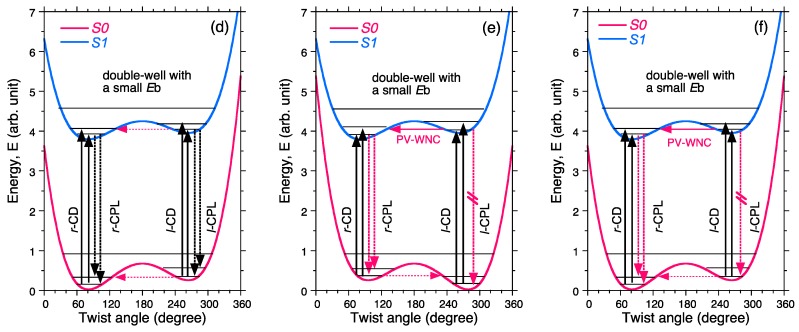
Potential energy surfaces of (**a**) an inherently achiral molecule in single-wells at the *S*_1_ and *S*_0_ states, (**b**) a rigid chiral molecule in mirror symmetric double-wells with a sufficiently large *E*_b_ value without tunneling at the *S*_1_ and *S*_0_ states, (**c**,**d**) a non-rigid chiral molecule in nearly mirror symmetric double-wells with small *E*_b_ values and resonance tunneling, causing parity-conserved mirror symmetric oscillations at the *S*_1_ and *S*_0_ states, and (**e**,**f**) a non-rigid chiral molecule in a nearly mirror symmetrical double-well at the *S*_1_ state with a small *E*_b_ and resonance tunneling in the presense of a handed weak neutral current, causing parity-violated non-mirror symmetric oscillation, while in a nearly mirror symmetrical double-well at the *S*_0_ state with a small *E*_b_ and and resonance tunneling in the absense of the weak neutral current, causing parity-conserved mirror symmetric oscillation. Red dotted arrow means parity-conserved resonance tunneling, while red solid denotes a handed parity-violating weak neutral current origin resonance tunneling.

The *E*_b_-*T*_rac_ relationship, as demonstrated in previous sections, can be categorized in three cases: (1) no detectable CD or CPL signals at the *S*_1_ and *S*_0_ states due to inherent molecular achirality, exemplified by unsubstituted acenes and pyrene (Scheme 1); (2) thermally and photophysically stable CD and CPL signals at the *S*_1_ and *S*_0_ states due to rigid chiral structures with a high *E*_b_ value exemplified by *D*-/*L*-camphor and *C*_2_-symmetric *R*-13/*S*-13 (Scheme 3); (3) no detectable and/or very weak CD signals at the *S*_0_ state, but noticeable CPL signals at the *S*_1_ state due to non-rigid enantiomers with a low *E*_b_ value which have rotational and flip-flop motional freedoms, as exemplified by structures in Schemes 2, 4–8, and *C*_2_-symmetric, but rotatable *R*-14/*S*-14 and *R*-15/*S*-15 in Scheme 3. The potential energy surfaces at the *S*_1_ and *S*_0_ states are illustrated as follows:

Case 1. Molecules in a single-well; no CD and no CPL signals are observable due to inherently achiral geometry at the *S*_0_/*S*_1_ states (Figure 15a).
Case 2. Rigid chiral molecules enforced by a stereocenter in a DW with sufficiently high *E*_b_ at the *S*_0_/*S*_1_ states; for these, if a single *(S)*-enantiomer were obtained, the CD and CPL signals would be mirror-images of the *(R)*-enantiomer due to the inherent chiral geometry of the *S*_0_/*S*_1_ states (Figure 15b). The value of *g*_lum_ is similar to that of *g*_abs_ and no change in sign between CPL and CD is expected. The stereocenter is responsible for the parity-conserving electromagnetic force.
Case 3. Non-rigid chiral molecules in a DW with sufficiently small *E*_b_ at the *S*_0_/*S*_1_ states; because the *(S)*-enantiomer is difficult to isolate due to rapid racemization by quantum tunneling, it is easy to be convinced that no CD or CPL signals at the *S*_0_/*S*_1_ states can be detected, and this is the conventional wisdom among chemists and described in textbooks on stereochemistry (Figure 14d and Figure 15c). The non-rigid chiral molecules are thus called CD-silent/CPL-silent molecules. The silence arises from quantum tunneling at the *S*_0_/*S*_1_ states. However, if the tunneling at the *S*_1_ state is a one-way event due to the *PV-WNC*, one can observe (–)-CPL (*right-handed* CPL only) (Figure 15e,f). In this case, the rapid left-right oscillation at the *S*_1_ state by quantum tunneling results in no detectable CD signals.

Open questions remain as to why the solvent viscosity discontinuously affects the (–)-CPL magnitude and the major role of the solvent viscosity. To control the resonance tunneling characteristics, the *E*_b_ value is a continuous variable, but can modify the resonance characteristics because of its exponential characteristics (Equation (1)). A higher viscosity increases the *E*_b_ value, resulting thereby in a smaller *ΔE*_±_ value. Conversely, a lower viscosity decreases the *E*_b_ value, affording a larger *ΔE*_±_ value. The former results in slower *T*_rac_ and the latter in faster *T*_rac_. To understand whether resonance tunneling between two sub-levels occurs, the discontinuous transition as a function of continuous external variables, like temperature, electric field, magnetic field and possibly solvent viscosity, could be evidence. Representative examples are superconductivity revealing a second-order phase transition as a function of temperature [95], integer-or-fractional quantum Hall effects as a function of external magnetic field and the current-voltage characteristics of hetero-junctions with double-wells/triple-barriers in AlGaAs/GaAs/AlGaAs/GaAs/AlGaAs devices [96].

Previously, we reported that several non-rigid chain-like Si-Si bond polymers (so-called helical polysilanes) which maintain rotational freedom of the Si-Si backbone in isooctane reveal discontinuous step-like helix-helix transition characteristics as a function of thermal energy bias. These helical polysilanes exhibited preferential (–)-sign CD signals in the magnitudes at most ~10 % below the helix-helix coalescence temperature (*T*_c_) regardless of the (*S*)- or (*R*)-chirality of the 3,7-dimethyloctyl side chains [58,59]. Similarly, in all cases of non-rigid luminophoric enantiomers, step-like and/or discontinuous transition behavior of (–)-CPL signals as a function of solvent viscosity could be evidence of unidirectional resonance tunneling induced by the *PV-WNC*. The greatest *g*_lum_ value (−1.5 × 10^−3^) (Figure 13b) corresponds to 0.075 % excess of right-handed circular polarization of light over left-handed (as seen from the observer).

The origin of the negative sign could be due to the postulated WNC with the universal handedness and is similar to the observed sign in APV experiments. According to PV-WNC force ideas, parity-odd rotational motion enforces both (*R*)- and (*S*)-forms in the same direction clockwise (or counterclockwise) facilitating radiation of (–)-sign CPL. On the other hand, the parity-conserved parity-even *EM* force allows multiple C-C bonds in the *(R)-*form to rotate with clockwise motion, and conversely, those with the *(S)-*form to rotate counterclockwise or *vice versa*. These motional dynamics are mirror symmetric. From our experiences, we can group the apparent CPL and CD spectral characteristics with their signs, magnitudes and wavelengths into four categories:

(i) Case 1. The value of *E*_b_ between rigid enantiomers is sufficiently high, for example, > 30 kcal mol^−1^ in the *S*_0_ and *S*_1_ state. In these cases, mirror-image CD and CPL spectra are apparent for the enantiomers. The parity-conserved (left-right equality or parity-even operational EM term) force is a deterministic factor. Such examples are *D*-10/*L*-10 and (*R*)-11/(*S*)-11 (Figure 3a–d).

(ii) Case 2. When 10 < *E*_b_ < 30 kcal mol^−1^ at the *S*_0_ and *S*_1_ states, one can observe non-mirror-image CPL and CD spectra. Although (+)- and (−)-signs in CPL and CD are primarily determined by molecular point and atrope chirality, the absolute magnitudes and wavelengths at vibronic CPL and CD bands are significantly different from each other. Such CPL spectra can be seen in (*R*)-12/(*S*)-12 dissolved in alkanols (Figure 3i–l) and *R*)-13/(*S*)-13 in *n*-pentane (Figure 3m). Several papers have been published describing such non-mirror-image CPL and CD spectra [58,59,60,71,87].

(iii) Case 3. When 1 < *E*_b_ < 10 kcal mol^−1^ at the *S*_1_ and *S*_0_ states, only (–)-sign CPL and (–)-sign CD spectra were observed. The parity non-conserved weak force is a determining factor at the *S*_1_ and *S*_0_ states. No such examples revealing only (–)-sign CPL associated with (–)-sign CD spectra have been reported yet.

(iv) Case 4. When 0 < *E*_b_ < 1 kcal mol^−1^ at the *S*_1_ and *S*_0_ states, no CD bands were observed, although (–)-sign CPL was apparent. Parity-non-conserved (left-right inequality or parity-odd operational weak force) is deterministic factor in the *S*_1_ state, while the parity-conserved force (parity-even EM term permitting left-right equality) is deterministic in the *S*_0_ state. The value of 1 kcal mol^−1^ is arbitrary but representative for the rotational barrier of single bonds. Many examples of such cases are described in this paper.

Indeed, our experimental demonstration here might be a possible answer to the greatest mystery of the L-R preference on Earth based on the *MPV* hypothesis inferred by *PV-WNC* scenario. The key is to utilize a series of well-defined fluorophores available commercially in achiral solvents. This idea enables anyone to reproduce our results using a well-maintained commercial or home-made CPL spectrometer and associated CD spectrometer at any place at any time. We eagerly await further theoretical studies treating the time-evolutional behavior of photoexcited molecular systems.

Other cogent arguments for the L-R preference in the framework of the parity-conserved *EM* force and cosmological scale origins are possible. The photoexcited handedness of non-rigid molecules in a small % *ee* should increase to ~100% *ee* in the ground state according to several nonlinear amplification scenarios [97,98,99,100,101,102,103,104,105,106,107], for example, autocatalytic self-replication [97,98], sergeant-and-soldier and majority rule polymer cases [99], polymerization [100] and helix-helix transitions [101]. In addition to the fundamental question of charge-parity-time criteria (the so-called, conservation of CPT theorem) [102], anti-neutrinos (left-handed as seen from the observer) of cosmological origin interacting with ^14^N in molecular clouds in star-forming regions of supernovae and neutron stars [103,104], parity violation of gravitational origin [105,106] and thermodynamic swirling flows in the north and south hemispheres on Earth [107] are of particular interest and might deserve to be used to verify these hypotheses in the future.

## 4. Materials and Methods

Instrumentation details, lists of luminohores and solvents associated with vendors [63,64,65], preparation of sample solutions and chiroptical analytical data [71,108] are as below:

### 4.1. Instrumentation

The CD and UV-visible spectra of the solution were recorded simultaneously at room temperature using a JASCO (Tokyo, Japan) J-820 spectropolarimeter using a cylindrical quartz cuvette with path lengths of 10 mm at ambient temperature. The cylindrical cuvette guaranteed a precision CD measurement compared to rectangular cuvettes that were often used in routine experiments. To obtain the precision CD/UV-visible spectra, a scanning rate of 50 and 100 nm min^−1^, a bandwidth of 2 nm, a response time of 2 s and 1 or 2 accumulations were employed. The instrument was fully aging at least 2 h to minimize unknown drifts of power supply and light source. Similarly, CPL and PL spectra were collected on a JASCO CPL-200 spectrofluoropolarimeter (Hachioji, Tokyo, Japan) using the cylindrical quartz cuvette with path lengths of 10 mm at room temperature. The optimal experimental parameters were that scanning rate: 20–50 nm min^−1^; bandwidth: 10 nm for excitation and detection; response time of PMT: 8–16 s during measurements and 2-to-8 accumulations. 

### 4.2. Lists of Materials

#### 4.2.1. Luminophores (vendor)

Section 1: naphthalene (Sigma-Aldrich, St. Louis, MO, USA), anthracene (FUJIFILM Wako Pure Chemical Corporation, Osaka, Japan), tetracene (Tokyo Chemical Company (TCI), Tokyo, Japan), pyrene (Wako).

Section 2: 9,10-diphenylanthracene (Sigma-Aldrich), 9,10-bis(phenylethynyl)anthracene (TCI), 2,7-di-*tert*-butyllpyrene (TCI), 1,3,6,8-tetraphenylpyrene (TCI), 5,12-bis(phenylethynyl)tetracene (Sigma-Aldrich).

Section 3: *D*-/*L*-camphor (Sigma-Aldrich), (*R*)-/(*S*)-(-)-1,1′-binaphthyl-2,2′-diyl hydrogen phosphate (TCI), (*R*)-/(*S*)-2,2′-dimethoxy-1,1′-binaphthyl (TCI), (*R*)-/(*S*)-2,2′-bis(methoxymethoxy)- 1,1’-binaphthyl (TCI).

Section 4: biphenyl (TCI), *p*-terphenyl (TCI), *p*-quaterphenyl (TCI), *p*-quinquphenyl (TCI), *p*-hexaphenyl (TCI), DMT (Exciton, Tokyo Instruments Inc (Tokyo, Japan)), Japanese vendor)), TMQ (Exciton), QUI (Exciton), Exalite 360 (Exciton), TBS (Exciton), [12] cycloparaphenylene (Kanto Chemicals, Tokyo, Japan).

Section 5: 9,9-dimethylfluorene (TCI), 9,9-dioctylfluorene (TCI), Exalite384 (Exciton), 9,9-dihexylfluorene trimer (American Dye Source (ADS), Quebec, Canada), 9,9-dihexylfluorene pentamer (ADS), 9,9-dihexylfluorene heptameter (ADS), Exalite 376 (Exciton), Exalite 404 (Exciton), Exalite 428 (Exciton), Exalite 416 (Exciton).

#### 4.2.2. Solvents [Vender, Viscosity in *cP* (Temperature in °C)

(1) *n*-alkanes (in order of viscosity): *n*-pentane (FUJIFILM Wako, 0.21 (25)), *n*-hexane (FUJIFILM Wako, 0.30 (25)), *n*-heptane (Sigma-Aldrich, 0.39 (25)), *n*-octane (Sigma-Aldrich, 0.51 (25), *n*-nonane (Sigma-Aldrich, 0.71 (20)), *n*-decane (Sigma-Aldrich, 0.85 (25)), *n*-undecane (Sigma-Aldrich, 0.93 (20)), *n*-dodecane (Sigma-Aldrich, 1.36 (25), *n*-tridecane (Sigma-Aldrich, 1.88 (20)), *n*-tetradecane (Fluka, 2.08 (25), *n*-pentadecane (Sigma-Aldrich, 2.86 (20)), *n*-hexadecane (Sigma-Aldrich, 3.71 (20))

(2) branched and cyclic alkanes (in order of viscosity): isooctane (Dotite, 0.50 (25)), cyclohexane (Dotite, 0.93 (22)), squalane (2,6,10,15,19,23-hexamethyltetracosane) (Sigma-Aldrich, 29.50 (25))

(3) Non-branched and *n*-alkanols (in order of viscosity): methanol (FUJIFILM Wako, 0.55 (25)), ethanol (FUJIFILM Wako, 1.09 (25)), *n*-propanol (Sigma-Aldrich, 1.96 (25)), *n*-butanol (FUJIFILM Wako, 2.59 (25)), *n*-pentanol (Sigma-Aldrich, 3.47 (25)), *n*-hexanol (Sigma-Aldrich, 4.59 (25)), *n*-heptanol (Wako, 5.97 (25)), *n*-octanol (Wako, 7.59 (25)), *n-*nonanol (Sigma-Aldrich, 9.51 (25)), *n*-decanol (Sigma-Aldrich, 11.50 (25)), ethylene glycol (FUJIFILM Wako, 16.1 (25), *n*-undecanol (Sigma-Aldrich, 16.95 (25)), 1,3-propandiol (FUJIFILM Wako, 33.0 (25)), 1,4-butanediol (FUJIFILM Wako, 71.0 (25))

(4) branched alkanols (in order of viscosity): isopropanol (Dotite, 2.07 (25), isobutanol (Sigma-Aldrich, 3.38 (25)), isopentanol (Sigma-Aldrich, 3.86 (25))

(5) chlorinated hydrocarbons (in order of viscosity): dichloromethane (Dotite, 0.41 (25)), chloroform (Dotite, 0.55 (25))

(6) Other solvents (in order of viscosity): diethyl ether (FUJIFILM Wako, 0.22 (25)), acetone (FUJIFILM Wako, 0.31 (25), acetonitrile (FUJIFILM Wako, 0.34 (25)), tetrahydrofuran (Dotite, 0.46 (25)), benzene (FUJIFILM Wako, 0.60 (25)), *p*-dioxane (Dotite, 1.10 (25)), anisole (TCI, 1.09 (25)), sulfolane (TCI, 10.10 (25)).

### 4.3. Preparation of Sample Solutions

First, a representatively stock solution (10^−3^ M) dissolved in spectroscopic grade CHCl_3_ (Dotite, Kumamoto, Japan) of luminophore was prepared. A small portion of the stock solution was injected to 1.9–2.1 mL of the desired liquid placed in the cylindrical quartz cuvette using a microsyringe, followed by manually shaking the mixture. Then, CPL/PL and CD/UV-vis spectra were measured.

### 4.4. Chiroptical Analysis

The dissymmetry factor of circular polarization at the S_0_ state (*g*_abs_) was theoretically calculated as *g*_abs_ = (*ε*_L_ − *ε*_R_)/[1/2(*ε*_L_ + *ε*_R_)], where *ε*_L_ and *ε*_R_ are the extinction coefficients for left- and right-CP light, respectively. The dissymmetry factor of circular polarization at the S_1_ state (*g*_lum_) was calculated as *g*_lum_ = (*I*_L_ − *I*_R_)/[1/2(*I*_L_ + *I*_R_)], where *I*_L_ and *I*_R_ are the output signals for left- and right-circularly polarized light under the unpolarized incident light, respectively. The parameter *g*_abs_ was experimentally determined using the expression. 

*Δε*/*ε* = [ellipticity (in mdeg)/32,980]/absorbance at the CD extremum, similar to the parameter *g*_lum_, which was calculated as *ΔI/I* = [ellipticity (in mdeg)/(32,980/ln10)]/[total PL intensity (in Volts)] at the CPL extremum.

## 5. Conclusions

We tested whether non-rigid, *π*-conjugated fluorophores in the *S*_1_ and *S*_0_ states in various achiral liquids with [*η*] ranging from 0.22 *cP* to 71.0 *cP* are optically inactive and mirror symmetrical by CPL and CD spectroscopy. The non-rigid luminophores included ten oligofluorene derivatives, eleven linear/cyclic oligo-*p*-phenylene derivatives, three binaphthyl derivatives, five fused aromatic rings (naphthalene, anthracene, tetracene, pyrene) with and without rotatable substituents. For comparison, we also tested unsubstituted naphthalene, anthracene, tetracene and pyrene as achiral planar fluorophors and a pair of camphor enantiomers as rigid chiral bicyclic fluorophors. We confirmed that the achiral rigid aromatic luminophores guaranteed no detectable CPL or CD signals and that camphor showed exactly mirror-image CPL and CD signals. 

On the other hand, without exception, all the non-rigid fluorophoric enantiomers showed (–)-sign CPL signals emission from the vibronic photoexcited state, in support of the molecular parity-violating hypothesis according to the *Z*^0^-boson origin *PV*-weak neutral current mechanism. Emission from these fluorophores increased *progressively*, *discontinuously* to ≈ −(0.2 to 2.0) × 10^−3^ as a function of solvent viscosity. We believe that this behavior is as a consequence of satisfying the *E*_pv_ ~ *E*_±_ condition associated with resonance quantum tunneling. In a series of oligofluorenes, the *g*_lum_ value extrapolated to zero-viscosity afforded a non-zero *g*_lum_ value, implying that the photoexcited oligofluorenes should emit (–)-sign CPL signals under collision-free vacuum conditions. 

We found that an unpolarized low-energy (3–5 eV) light source generated handed chiral molecular species as observed by the emission of (–)-sign circularly polarized light in the UV-visible region when non-rigid racemic molecules with low barrier heights tested in this work were dissolved in achiral liquids, and even under collision-free vacuum conditions. Circularly and linearly polarized light sources may be not crucial.

## Supplementary Materials

The following are available online, Figure S1: CPL/PL and CD/UV-vis spectral characteristics of pseudo-*D*_2h_-symmetrical fused aromatics carrying multiple rotatable substituents (**6**–**9**), Figure S2: CD/UV-vis and CPL/PL spectra of rigid binaphthol derivatives (*R*-11 and *S*-11) and semirigid binaphthol derivatives (*R*-12 and *S*-12), Figure S3: CPL/PL and CD/UV-Vis spectra of unsubstituted linear and cyclic oligo-*p*-phenyls (Ph3, Ph4, Ph5 and 12-CPP), Figure S4: CD/UV-vis and CPL/PL spectra of substituted linear oligo-*p*-phenyls (DMT, Exalite360, TMQ, QUI and TBS), Figure S5: CPL/PL and CD/UV-vis spectra of FL1-C_1_ and FL1-C_8_, Figure S6: CPL/PL and CD/UV-vis spectra of FL2-C_3_ (Exalite 384), Figure S7: CPL/PL and CD/UV-vis spectra of FL3-C_6_ (fluorene trimer), Figure S8: CPL/PL and CD/UV-vis spectra of FL5-C_6_ (fluorene pentamer), Figure S9: CPL/PL and CD/UV-vis spectra of FL7-C_6_ (fluorene heptamer), Figure S10: Raw CPL/PL spectra of **2**, *R*-11 and *S*-11 in chloroform and Exalite 428 in *n*-undecanol conducted by JASCO company at Tokyo with a CPL-300 spectrometer (16 November 2018 and 24 November 2018), Figure S11: Materials and methods including instrumentation, lists of materials, preparation of sample solutions and chiroptical analysis.
