# Correction: Fujiki, M., et al. Questions of Mirror Symmetry at the Photoexcited and Ground States of Non-Rigid Luminophores Raised by Circularly Polarized Luminescence and Circular Dichroism Spectroscopy: Part 1. Oligofluorenes, Oligophenylenes, Binaphthyls and Fused Aromatics. *Molecules,* 2018, *23*, 2606

**DOI:** 10.3390/molecules23123348

**Published:** 2018-12-18

**Authors:** Michiya Fujiki, Julian R. Koe, Takashi Mori, Yoshihiro Kimura

**Affiliations:** 1 Division of Materials Science, Graduate School of Science and Technology, Nara Institute of Science and Technology (NAIST), 8916-5 Takayama, Ikoma, Nara 630-0036, Japan; mori@tri-osaka.jp (T.M.); yoshi19791024uk@gmail.com (Y.K.); 2 Department of Natural Sciences, International Christian University (ICU), 3-10-2 Mitaka, Tokyo 181-8585, Japan

The authors wish to make the following correction to their paper [1]:

We found that Figure 9g,h was lacking on the published page 19, though a full caption of Figure 9a–h was already given. The addition of Figure 9g,h does not affect the scientific results.

The manuscript will be updated and the original will remain online on the article webpage. The authors would like to apologize for any inconvenience caused to the readers by these changes.

## Figures and Tables

**Figure 9:**
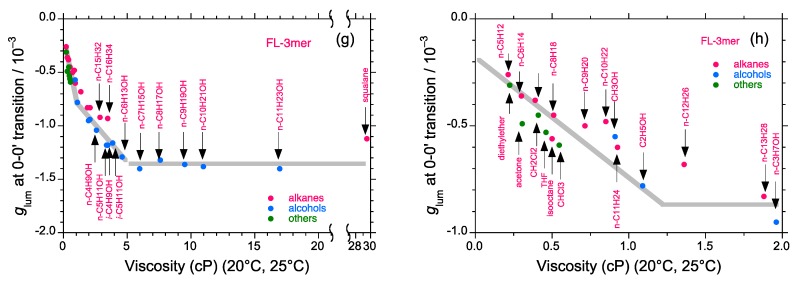
CPL/PL spectra of FL3-C_6_ (fluorene trimer) excited at 330 nm at room temperature; (**g**) the *g*_lum_ value at the 0-0’ band of FL3-C_6_ as a function of viscosity (in *cP* at 20–25 °C) of various achiral alkanes, alkanols and other solvents and (**h**) magnification of the viscosity region 0–2.0 *cP*. Path length: 10 mm, cylindrical cuvette, conc.: 2 × 10^−5^ M.
