# Supplementary material for: Questions of Mirror Symmetry at the Photoexcited and Ground States of Non-Rigid Luminophores Raised by Circularly Polarized Luminescence and Circular Dichroism Spectroscopy: Part 1. Oligofluorenes, Oligophenylenes, Binaphthyls and Fused Aromatics
Source: Molecules. 2018 Oct 11;23(10):2606. doi: 10.3390/molecules23102606 (PMC6222818; doi:10.3390/molecules23102606)
Supplement: Supplementary file 1 [file molecules-23-02606-s001.pdf]

Supplementary Materials (SM) for

Questions of mirror symmetry at the photoexcited and ground states of non-rigid luminophores raised by circularly polarized luminescence and circular dichroism spectroscopy. Part 1. Oligofluorenes, oligophenylenes, binaphthyls, and fused aromatics

Michiya Fujiki [1,*], Julian R. Koe [2,*], Takashi Mori [1] and Yoshihiro Kimura [1]

1 Division of Materials Science, Graduate School of Science and Technology, Nara Institute of Science and Technology (NAIST), 8916-5 Takayama, Ikoma, Nara 630-0036, Japan; mori@tri-osaka.jp (T.M.); yoshi19791024uk@gmail.com (Y.K.)

2 Department of Natural Sciences, International Christian University (ICU), 3-10-2 Mitaka, Tokyo, 181-8585, Japan

* Correspondence: fujikim@ms.naist.jp (M.F.); koe@icu.ac.jp (J.R.K.); Tel.: +81-743-72-6040 (M.F.); +81-422-33-3249 (J.R.K.)

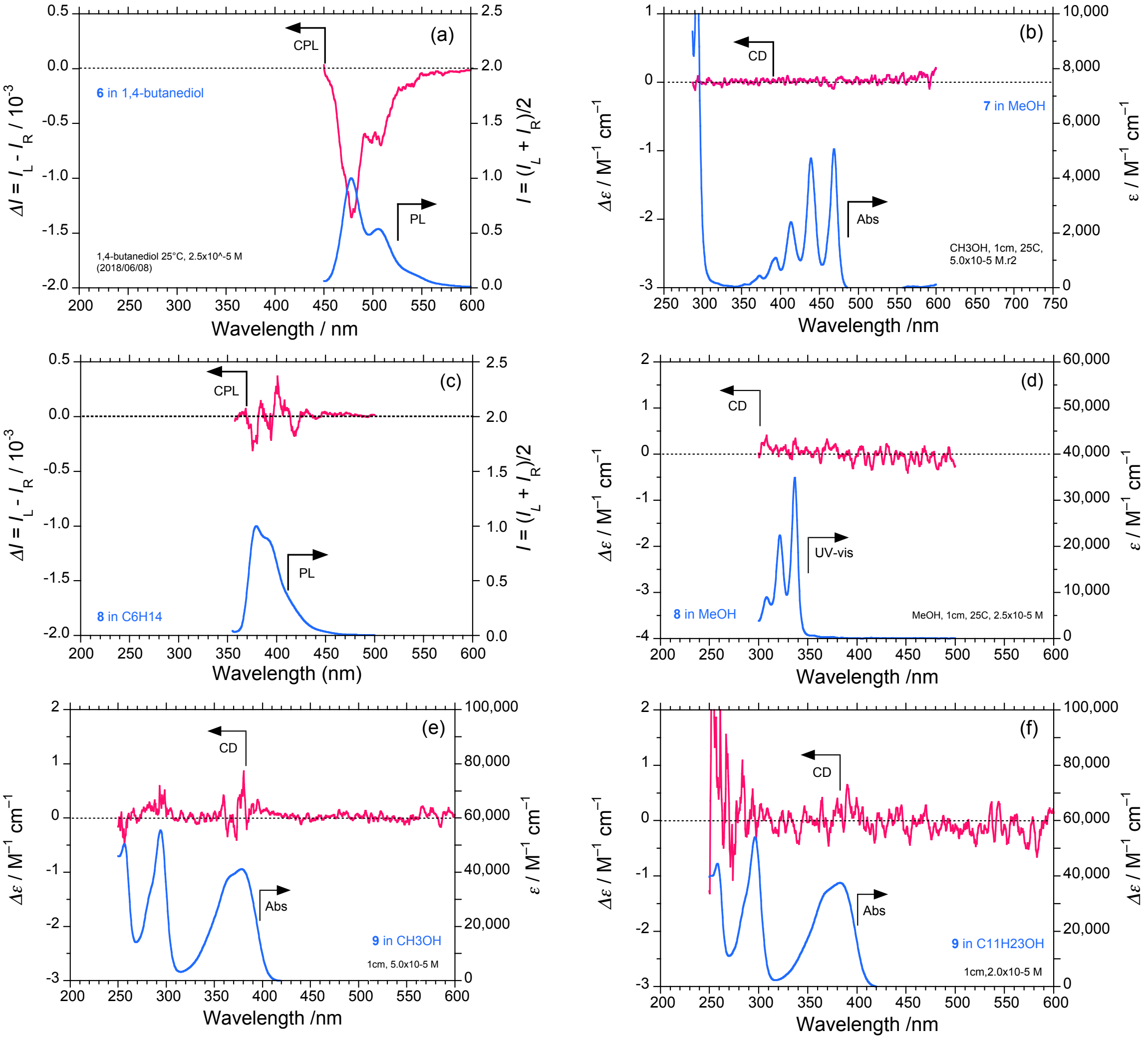

Figure S1. CPL/PL and CD/UV-vis spectral characteristics of pseudo-D_{2h}-symmetrical fused aromatics carrying multiple rotatable substituents in various liquids at room temperature. (a) CPL/PL spectra of **6** excited at 320 nm in 1,4-butanediol. (b) CD/UV-vis spectra of **7** in methanol. (c) CPL/PL spectra of **8** excited at 325 nm in *n*-hexane. (d) CD/UV-vis spectra of **8** in methanol. CD/UV-vis spectra of **9** in (e) methanol and (f) *n*-undecanol. Measurement conditions: path length: 10 mm, cylindrical cuvette, conc.: (2.5–5.0) x 10^{-5} M at room temperature.

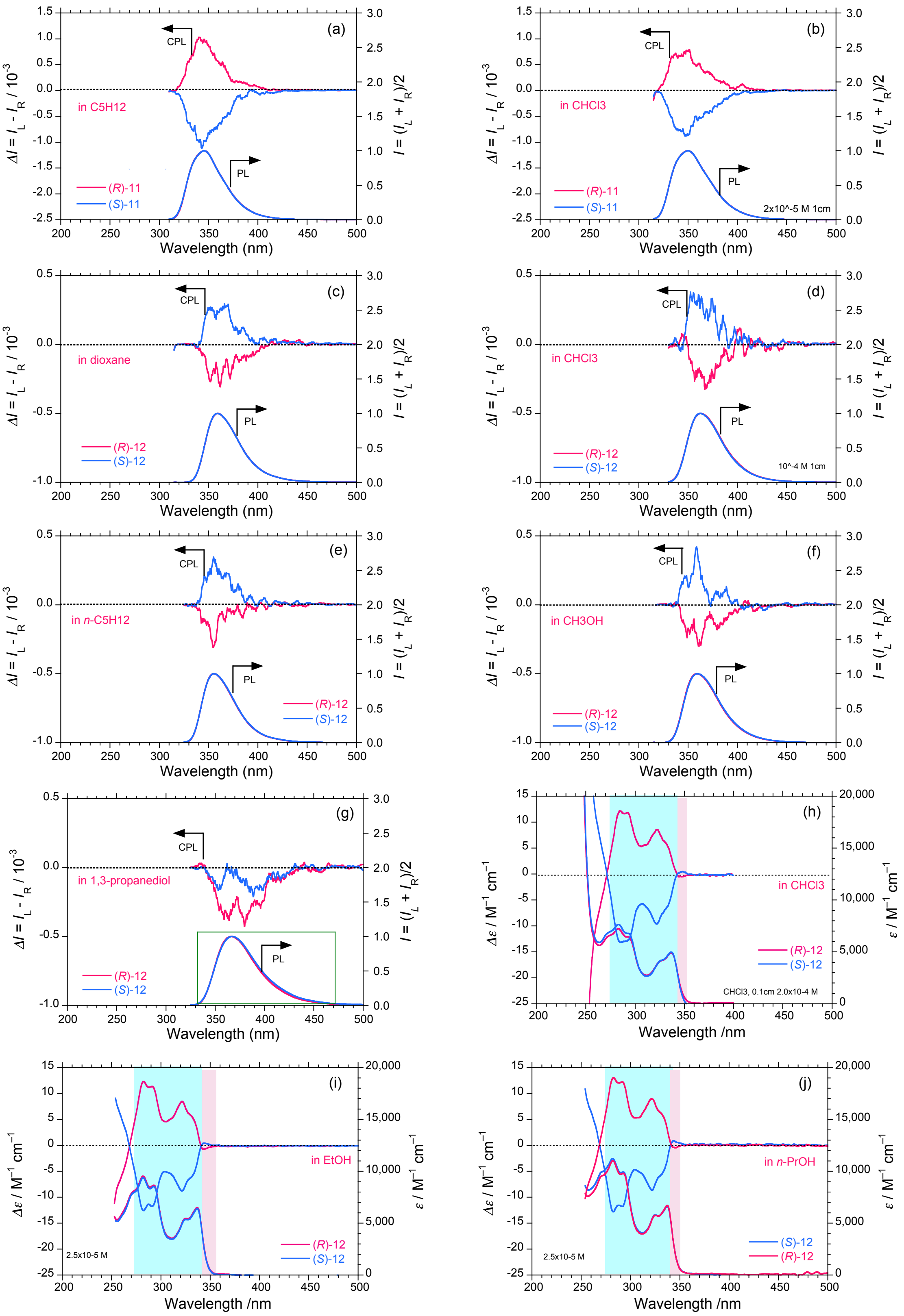

Figure S2. CD/UV-vis and CPL/PL spectra of rigid binaphthol derivatives and semirigid binaphthol derivatives allowing pivotal motion in various solvents at room temperature (path length: 10 mm, cylindrical cuvette, conc.: (2–5) x 10^{-4} M: CPL/PL spectra of ***R*-11** and ***S*-11** excited at 290 nm (a) in *n*-pentane and (b) in chloroform; CPL/PL spectra of ***R*-12** and ***S*-12** excited at 295 nm (c) in *p*-dioxane, (d) in chloroform, (e) in *n*-pentane, (f) in methanol, and (g) in 1,3-propandiol; CD/UV-vis spectra of ***R*-12** and ***S*-12** (h) in chloroform, (i) in ethanol, and (j) in *n*-propanol.

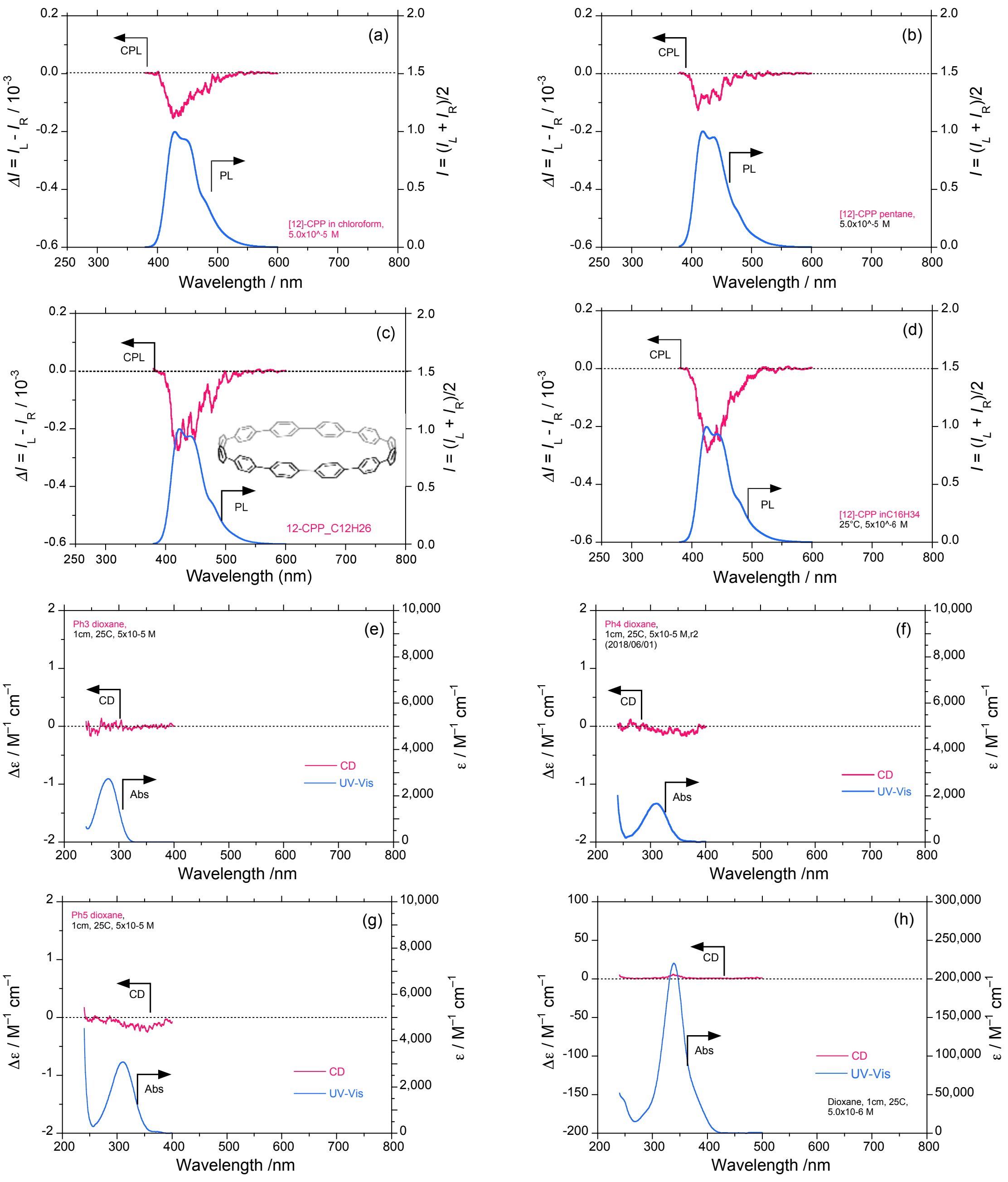

Figure S3. CPL/PL and CD/UV-Vis spectra of unsubstituted linear and cyclic oligo-*p*-phenyls in several solvents at room temperature (path length: 10 mm, cylindrical cuvette, conc.: (0.5–5) x 10^{-5} M. CPL/PL spectra of **12-CPP** excited at 350 nm (a) in chloroform, (b) in *n*-pentane, (c) in *n*-dodecane, and (d) in *n*-hexadecane; CD/UV-Vis spectra of (e) **Ph3**, (f) **Ph4**, (g) **Ph5**, **and** (h) **12-CPP** in *p*-dioxane.

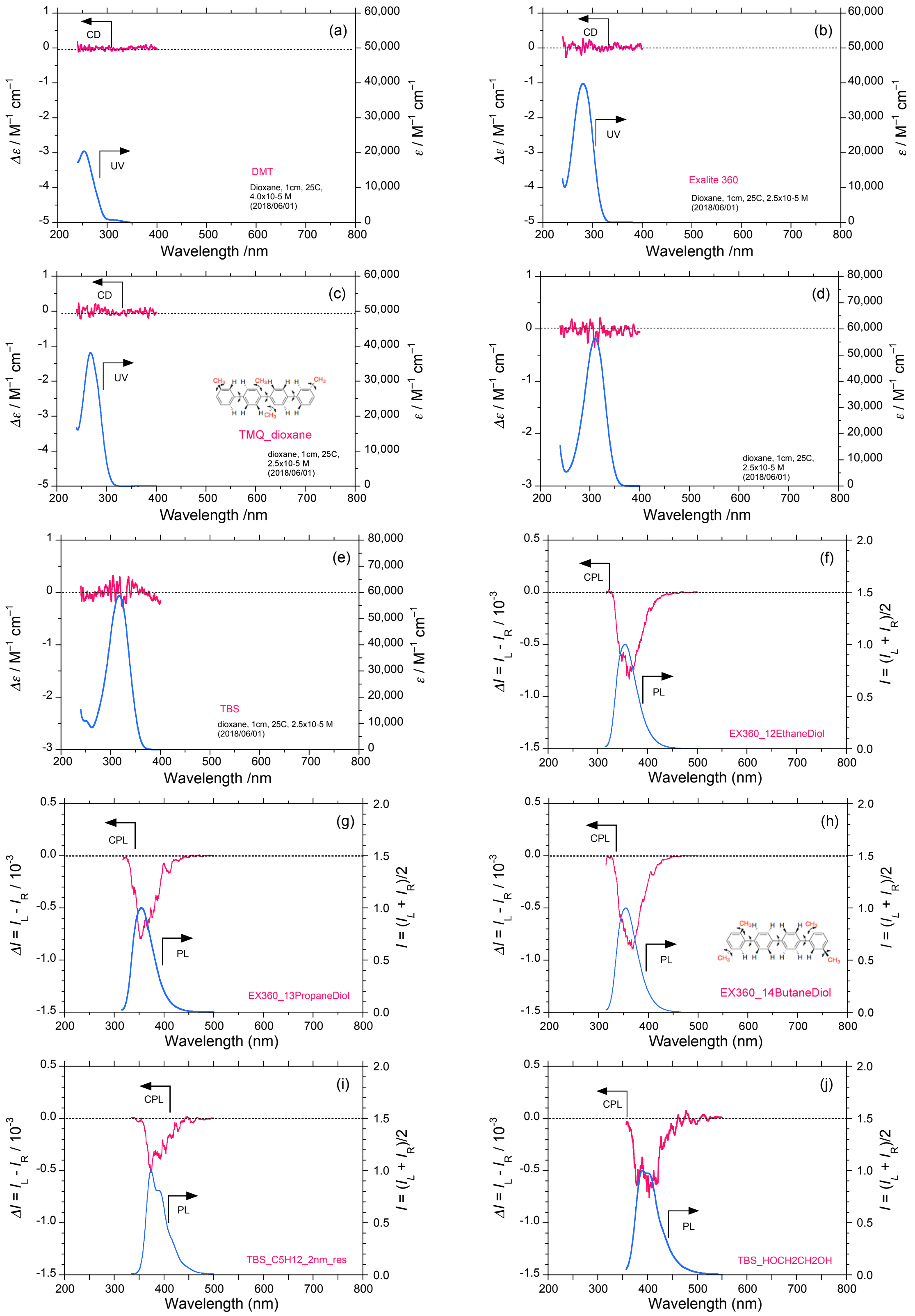

Figure S4. CD/UV-vis spectra of (a) **DMT**, (b) **Exalite360**, (c) **TMQ**, (d) **QUI**, and (e) **TBS** in *p*-dioxane. CPL/PL spectra of **DMT** excited at 315 nm. CPL/PL spectra of linear oligo-*p*-phenyls carrying methyl and *t*-butyl groups in *p*-dioxane and other solvents at room temperature; CPL/PL spectra of **Exalite360** excited at 280 nm (f) in ethylene glycol, (g) in 1,3-propane diol, and (h) in 1,4-butane diol; CPL/PL spectra of **Exalite360** excited at 280 nm (f) in ethylene glycol, (g) in 1,3-propane diol, and (h) in 1,4-butane diol; CPL/PL spectra of **TBS** excited at 315 nm (i) in *n*-pentane and (j) in ethylene glycol.

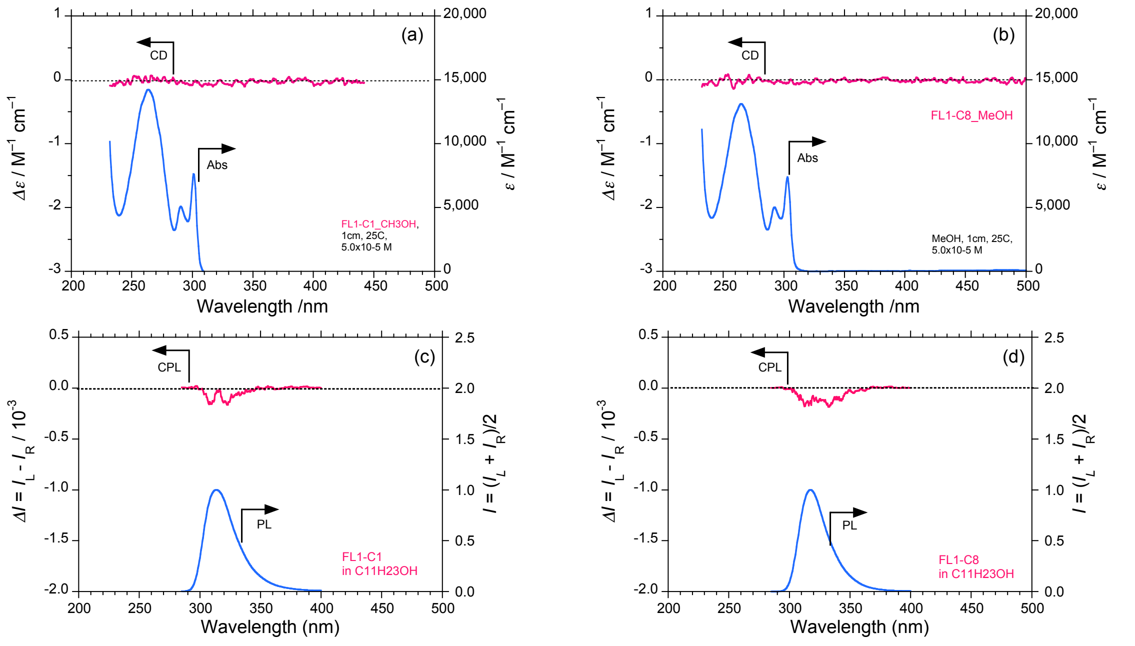

Figure S5. CD/UV-Vis spectra of (a) **FL1-C₁** and (b) **FL1-C₈** in methanol. CPL/PL spectra of (c) **FL1-C₁** excited at 255 nm and (d) **FL1-C₈** excited at 255 nm at room temperature in *n*-undecanol. Path length: 10 mm, cylindrical cuvette, conc.: 5 x 10^{-5} M.

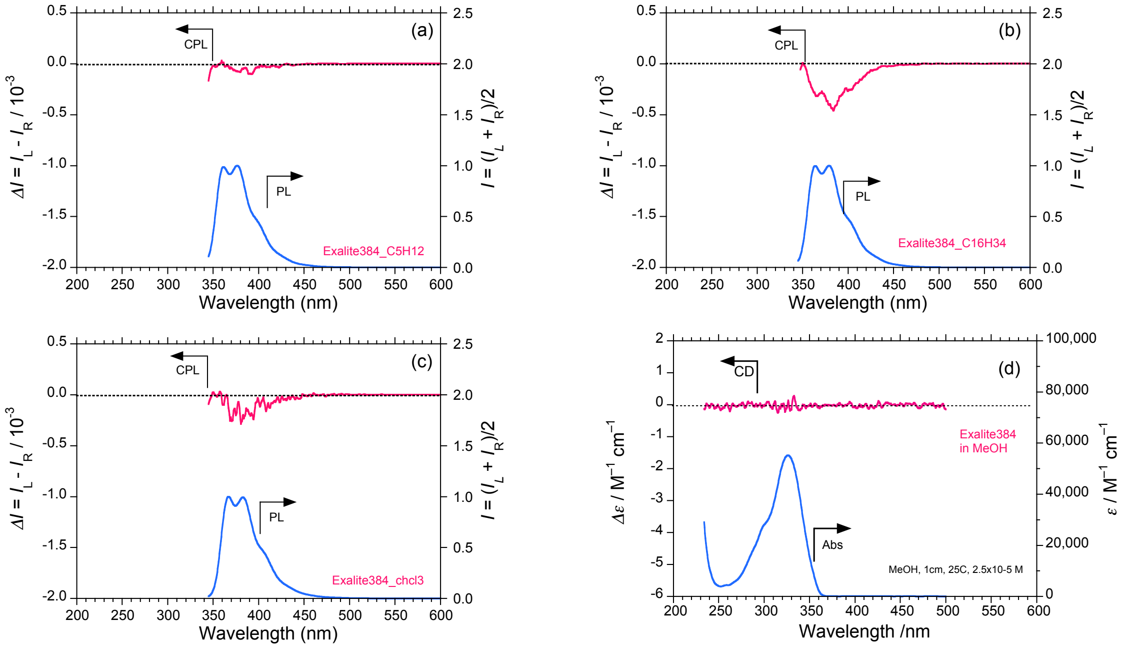

Figure S6. CPL/PL spectra of **FL2-C₃** (**Exalite 384**, fluorene dimer) excited at 315 nm at room temperature (a) in *n*-pentane, (b) in *n*-hexadecane, and (c) in chloroform. (d) CD/UV-vis spectrum of **FL2-C₃** (**Exalite 384**) in methanol. Path length: 10 mm, cylindrical cuvette, conc.: 1 x 10^{-5} M.

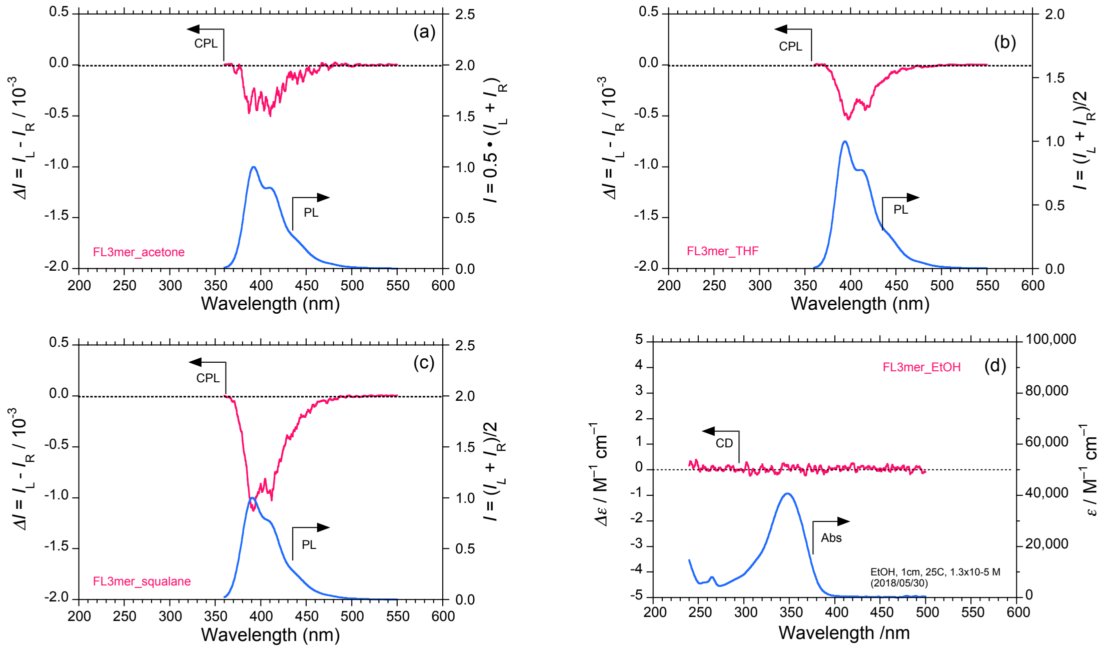

Figure S7. CPL/PL spectra of **FL3-C$_6$** (fluorene trimer) excited at 330 nm at room temperature (a) in acetone, (b) in tetrahydrofuran, and (c) in squalane; (d) CD/UV-vis spectrum of **FL3-C$_6$** in ethanol at room temperature. Path length: 10 mm, cylindrical cuvette, conc.: (1.3-2.0) x 10^{-5} M.

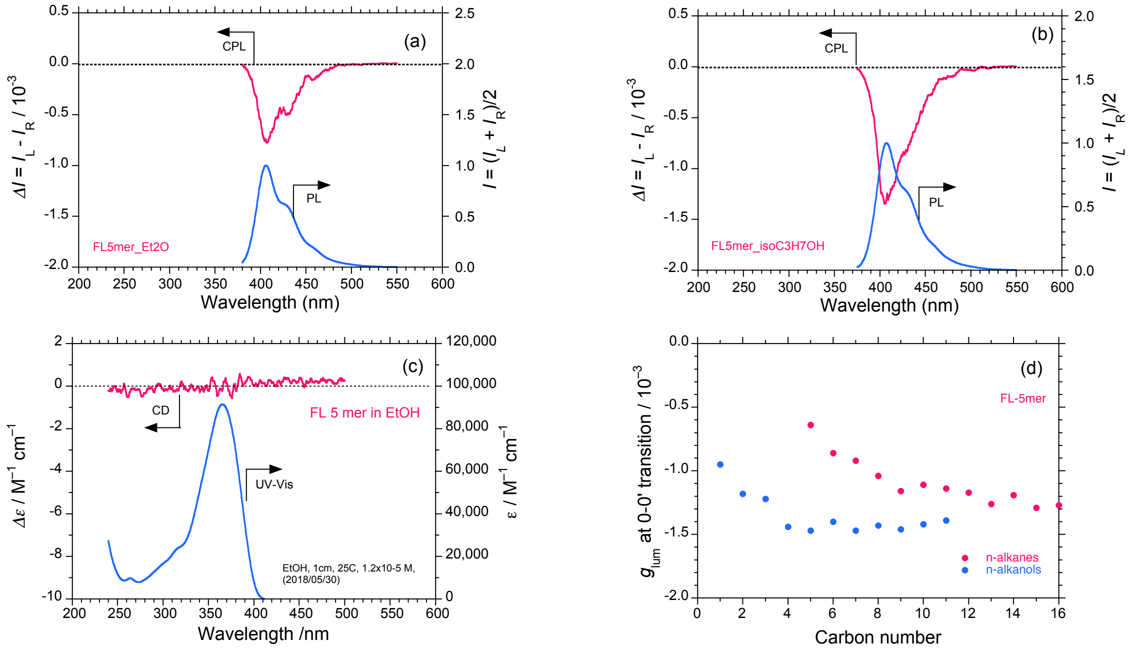

Figure S8 CPL/PL spectra of **FL5-C$_6$** (fluorene pentamer) excited at 340 nm at room temperature (a) in diethylether and (b) in isopropanol; (c) CD/UV-vis spectrum of **FL5-C$_6$** in ethanol at room temperature; (d) the g_{lum} value at 0-0′ band of **FL5-C$_6$** as a function of carbon numbers in two series of *n*-alkanes and *n*-alkanol including methanol and ethanol. Path length: 10 mm, cylindrical cuvette, conc.: 5 x 10^{-6} M.

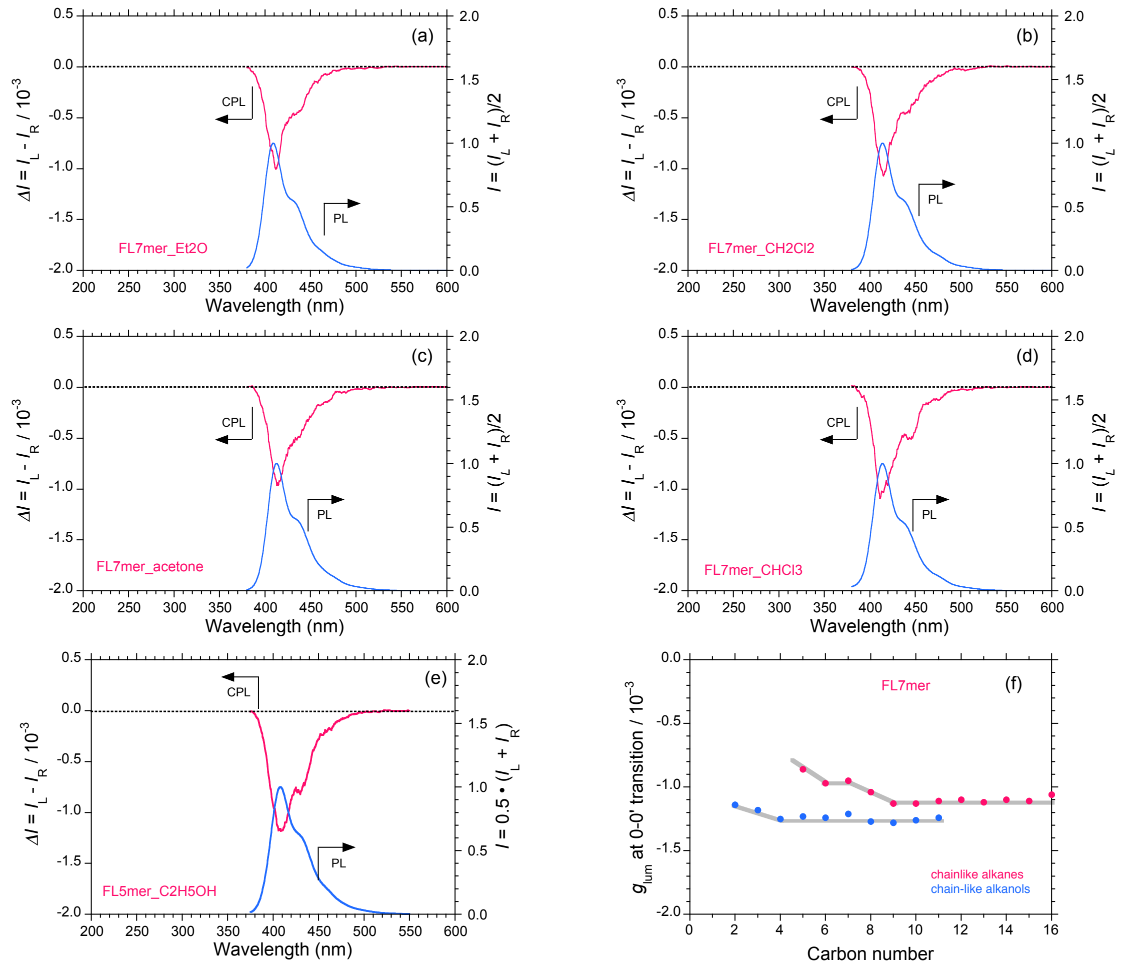

Figure S9. CPL/PL spectra of **FL7-C$_6$** (fluorene heptamer) excited at 350 nm at room temperature (a) in diethylether, (b) in dichloromethane, (c) in acetone, and (d) in chloroform. (e) CD/UV-vis spectra of **FL7-C$_6$** in ethanol at room temperature. (f) The g_{lum} value at 0-0′ band of **FL7-C$_6$** as a function of carbon numbers in two series of *n*-alkanes and *n*-alkanol including ethanol. Path length: 10 mm, cylindrical cuvette, conc.: 5 x 10^{-6} M.

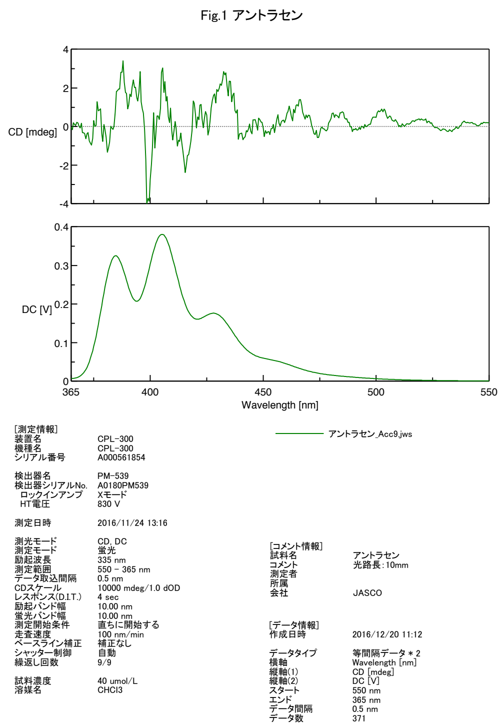

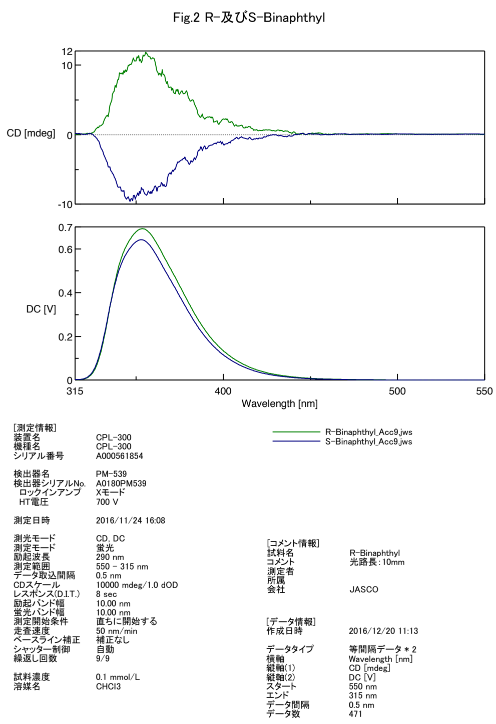

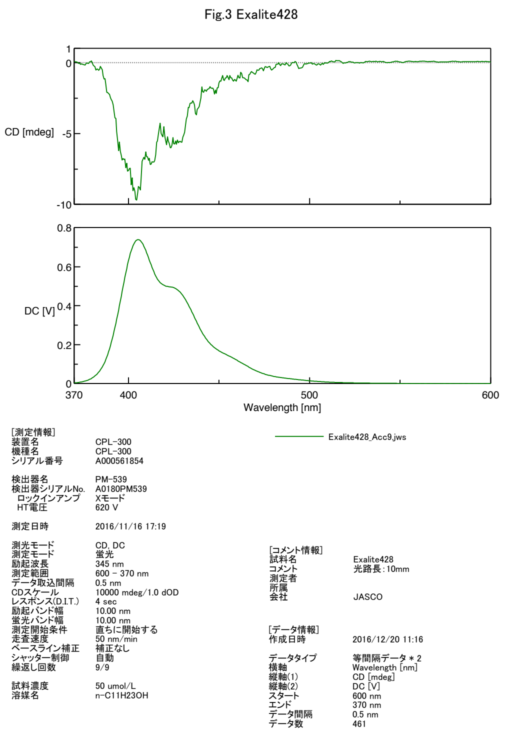

Figure S10. Raw CPL/PL spectra of **2** in chloroform, ***R*-11** and ***S*-11** in chloroform, and **Exalite 428** in *n*-undecanol at ambient temperature that were obtained independently by JASCO company using CPL-300 spectrofluoropolarimeter that is a newly designed model based on our CPL-200 spectrofluoropolarimeter (measured on 16 Nov 2018 and 24 Nov 2018).
